# Cobalt Single‐Atom Catalysts for Ultrafast Sulfamethoxazole Degradation: Unveiling the Chloride‐Ion‐Enhanced Formation of Co(IV)=O

**DOI:** 10.1002/advs.75549

**Published:** 2026-05-05

**Authors:** Anting Ding, Jing Lu, Yafei Fan, Chenyu Zeng, Yuhang Lin, Yufei Shi, Zulin Zhang

**Affiliations:** ^1^ Xianghu Laboratory Hangzhou China; ^2^ Department of Environmental Engineering College of Environmental & Resource Sciences Zhejiang University Hangzhou China; ^3^ Key Lab for Colloid and Interface Science of the Ministry of Education School of Chemical and Chemical Engineering Shandong University Jinan China; ^4^ The James Hutton Institute Craigiebuckler Aberdeen UK

**Keywords:** chloride ions, high‐valent cobalt, peroxymonosulfate, single‐atom catalysts, sulfamethoxazole

## Abstract

Chloride ions, widely regarded as radical scavengers, can paradoxically enhance pollutant degradation in a Co–N_4_ single‐atom catalyst (SAC) activated peroxymonosulfate (PMS) system. In the presence of Cl^−^, sulfamethoxazole (SMX) oxidation is dramatically accelerated via an HOCl‐mediated non‐radical route that promotes high‐valent Co(IV) = O generation, increasing the observed rate constant from 0.736 to 2.53 min^−^^1^ and enabling rapid, selective SMX removal with minimal matrix interference. In situ surface‐enhanced Raman spectroscopy and density functional theory calculations analyses confirm this Cl^−^‐enhanced pathway, with Co(IV) = O emerging as the predominant oxidant. We further translate this mechanism into a flexible polytetrafluoroethylene (PTFE)‐supported Co–N_4_ membrane reactor, which sustains high SMX removal under continuous flow with minimal hydraulic loss. Toxicity assays show significantly reduced ecotoxicity of degradation byproducts and confirm the treated effluent's biocompatibility. These findings establish a chloride‐augmented oxidation strategy that transforms a common wastewater constituent into a co‐promoter, demonstrating a robust, selective, and scalable platform for advanced water purification.

## Graphical abstract

An atomically dispersed Co–N_4_ single‐atom catalyst rapidly activates peroxymonosulfate to remove the antibiotic sulfamethoxazole from water. Common ions have little impact, but chloride dramatically speeds degradation by promoting a high‐valent Co(IV) = O oxidant via in situ HOCl–catalyst electron transfer.

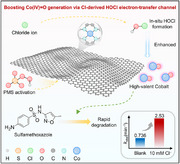

## 1 Introduction

Antibiotics are a major class of emerging contaminants (EC) that pose substantial ecological and public health risks owing to their environmental persistence, tendency for bioaccumulation, and contribution to the increasing prevalence of antimicrobial resistance [1, 2, 3, 4, 5]. Accordingly, peroxymonosulfate (PMS)‐based advanced oxidation processes have been widely explored for antibiotic removal, as they generate a diverse range of reactive oxygen species (ROS) that facilitate rapid and versatile oxidative degradation under environmentally relevant conditions [6, 7, 8, 9, 10]. Among the numerous PMS activation catalysts reported to date, single‐atom catalysts (SACs) have attracted significant attention because of their high atomic efficiencies, uniform active‐site structures, and exceptional catalytic tunabilities [11, 12, 13]. Specifically, SAC‐mediated PMS activation yields diverse ROS, including radicals (e.g., hydroxyl, sulfate, and superoxide radicals) and non‐radicals (e.g., ^1^O_2_ and high‐valent metal–oxo species) [14, 15, 16, 17]. However, the efficacy of the radical species is compromised by interference from anions and organic matter present in water, significantly reducing PMS utilization and potentially leading to the formation of undesirable halide byproducts [18]. In contrast, non‐radical pathways offer selective and robust oxidation mechanisms, especially for high‐valent metal–oxo species and ^1^O_2_, which facilitate selective contaminant degradation owing to their specificity, longevity, and resistance to matrix components [19, 20, 21]. SAC‐mediated non‐radical pathways have recently been recognized as more suitable than conventional radical routes for the treatment of complex wastewater. For example, Pei et al. demonstrated that an iodine‐based single‐atom catalyst (I–NC) activates PMS via a ^1^O_2_‐dominated non‐radical pathway, which exhibits remarkable resistance to coexisting anions, cations, and natural organic matter, while being tolerant to a wide pH range (3.1–10.9), thereby outperforming radical‐based PMS systems in complex water matrices [18].

More recently, Co‐based SACs (Co‐SACs) anchored on N‐doped carbon have emerged as prototypical PMS activators. These materials are distinguished by their tendency to selectively and efficiently generate long‐lived high‐valent Co(IV) = O species, which show strong anti‐interference behavior toward common anions and favor non‐radical PMS activation pathways over those involving short‐lived sulfate or hydroxyl radicals [22, 23, 24, 25, 26, 27, 28]. It should be noted that high‐valent metal‐mediated PMS activation is not limited to single‐atom catalysts. Earlier studies in homogeneous Co(II)/PMS systems provided compelling evidence for the involvement of Co(IV)‐oxo species, supported by probe reactions, isotope‐labeling analysis, and kinetic modeling [29]. Subsequent work further extended this mechanistic picture to molecular cobalt catalysts and heterogeneous non‐single‐atom materials, including cobalt phthalocyanine complexes and perovskite‐type oxides [30, 31], indicating that high‐valent metal‐oxo chemistry is a broader reaction motif in PMS activation rather than a phenomenon unique to SACs. Nevertheless, SACs remain especially valuable platforms for mechanistic investigation because their well‐defined coordination environments enable more precise correlation of local electronic structure with PMS adsorption, O─O bond cleavage, and high‐valent metal‐oxo formation [32]. Against this broader background, Co‐based SACs provide an ideal model system for clarifying how coexisting matrix ions regulate the generation and reactivity of Co(IV) = O under realistic water conditions. However, practical wastewater treatment is complicated by the presence of abundant inorganic anions (e.g., Cl^−^, HCO_3_^−^/CO_3_^2−^, NO_3_^−^, H_2_PO_4_^−^, and SO_4_^2−^) and natural organic matter, which either scavenge ROS or participate in secondary reactions that reshape persulfate‐activation pathways. Numerous studies have shown that chloride ions (Cl^−^) and natural organic matter can generate reactive chlorine species or quench radicals during persulfate/PMS‐based advanced oxidation, while carbonate and bicarbonate often suppress pollutant degradation by consuming SO_4_^•−^ and ^•^OH [33]. Emerging SAC studies have revealed that matrix ions exert similarly complex, ion‐specific effects; while Cl^−^, H_2_PO_4_^−^, and NO_3_^−^ negligibly affect the degradation kinetics of some Fe‐SAC/PMS or Fenton‐like systems, carbonate and humic substances markedly inhibit pollutant removal, which highlights the vulnerability of single‐atom‐mediated pathways to water chemistry [34]. However, the mechanisms by which typical coexisting ions in realistic matrices collectively regulate the PMS activation selectivity—especially the balance between radical species, non‐radical Co(IV) = O, and ions that act as genuine promoters— remain poorly understood in Co‐based SACs. This uncertainty hampers the rational design of Co‐SACs for use in Cl^−^‐rich wastewater‐treatment applications, thereby necessitating the systematic evaluation of ion‐specific effects and their mechanistic origins.

In this work, we reveal that ubiquitous chloride ions—traditionally regarded as radical scavengers in PMS‐based advanced oxidation—can be repurposed as a kinetic “promoter” to intensify non‐radical oxidation on a Co–N_4_ single‐atom catalyst. The PMS/CoN_4_ system enables rapid sulfamethoxazole (SMX) abatement through a high‐valent cobalt pathway, while chloride introduction further boosts the pseudo‐first‐order rate constant from 0.736 to 2.53 min^−^^1^. Mechanistic interrogation combining quenching experiments, electron paramagnetic resonance (EPR) measurements, in situ surface‐enhanced Raman spectroscopy, and density functional theory (DFT) calculations uncovers a Cl^−^‐enabled route in which in situ generated HOCl is preferentially intercepted by Co–N_4_ sites to accelerate Co(IV) = O formation, rather than acting as the terminal oxidant for nonspecific chlorination. Importantly, this chloride‐enhanced high‐valent regime is translated into a polytetrafluoroethylene (PTFE)‐confined catalytic membrane reactor, demonstrating robust performance under continuous‐flow operation. Overall, this work establishes a matrix‐ion‐enabled strategy that converts a common wastewater constituent into a co‐promoter for selective Co(IV) = O chemistry, offering a mechanistically grounded route toward practical and resilient PMS‐based water purification.

## 2 Results

### 2.1 Synthesis and Characterization

**FIGURE 1:**
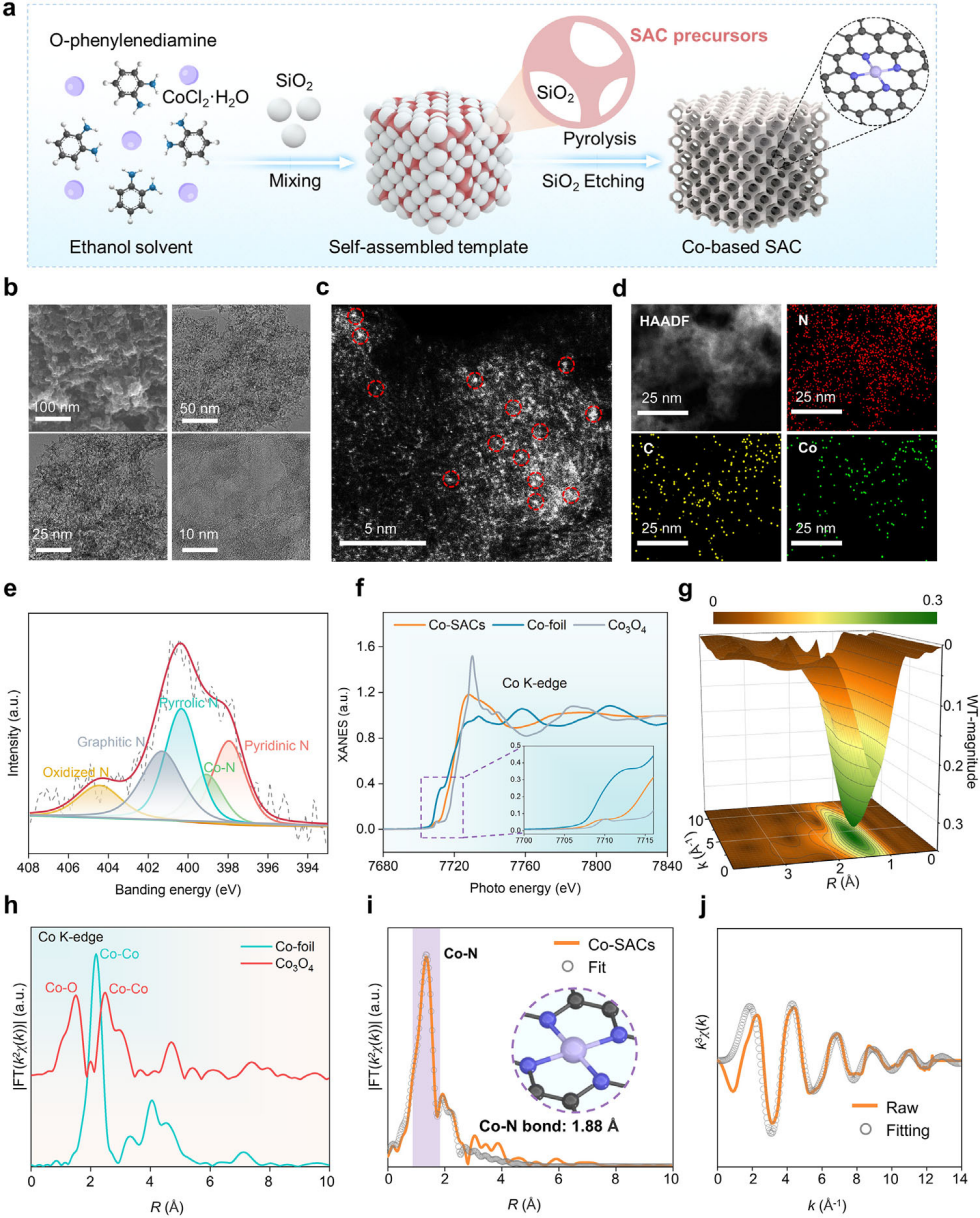
Atomic structure characterization of CoN_4_. (a) Schematic diagram of the synthesis of CoN_4_. (b) SEM (100 nm) and TEM (50, 25, and 10 nm) images of CoN_4_. (c) HAADF‐STEM image of CoN_4_ (5 nm), the single atoms are marked with red circles. (d) HAADF‐STEM image and corresponding EDS mapping of C, N, and Co. (e) N 1s XPS spectra of CoN_4_. (f) Normalized XANES spectra at the Co K‐edge of CoN_4,_ along with reference samples. (g) Wavelet transform analysis of Co K‐edge EXAFS data. (h) Fourier‐transformed (FT) magnitudes of the experimental Co K‐edge EXAFS signals of Co_3_O_4_ and Co foil. Fitting curves of the EXAFS of CoN_4_ in the *R*‐space (i) and *k*‐space (j).

Co‐SAC was synthesized via a two‐step process involving the formation of a cobalt–silica composite precursor followed by SiO_2_ template removal (see Text S2; Figure 1a). Morphological characterization by scanning electron microscopy (SEM) and transmission electron microscopy (TEM) revealed a well‐defined 3D honeycomb‐like architecture with interconnected mesopores (Figure 1b), which contributed to a high specific surface area of 944 m^2^ g^−^^1^ (Figure S1). Raman spectroscopy revealed an I_D_/I_G_ ratio (ratio of D‐ to G‐band intensities) of 0.99 (Figure S2), suggesting the coexistence of graphitic domains and abundant structural defects that can effectively anchor isolated Co centers [35]. Aberration‐corrected high‐angle annular dark‐field scanning transmission electron microscopy (HAADF‐STEM) imaging displayed numerous atomically bright spots homogeneously distributed on the carbon substrate (Figure 1c), confirming the presence of atomically dispersed Co atoms, as corroborated by the absence of crystalline Co‐related peaks in the powder X‐ray diffraction (XRD) pattern (Figure S2). Energy‐dispersive X‐ray spectroscopy (EDS) elemental mapping verified the uniform incorporation of nitrogen into the carbon lattice and its coordination with the isolated Co atoms (Figure 1d). The high‐resolution N 1s X‐ray photoelectron spectroscopy (XPS) spectrum of Co‐SAC exhibited five distinct peaks, corresponding to pyridinic N, Co–N, pyrrolic N, graphitic N, and oxidized N species [36, 37] (Figure 1e), collectively confirming the successful formation of atomically dispersed Co–N_4_ active sites within a defect‐rich carbon framework.

To gain deeper insight into the coordination and oxidation states of the Co centers in Co‐SAC, X‐ray absorption near‐edge structure (XANES) and extended X‐ray absorption fine structure (EXAFS) analyses were performed. As illustrated in Figure 1f, the absorption edge of CoN_4_ is located between those of the Co foil and Co_3_O_4_, suggesting that the Co species possess an intermediate oxidation state between 0 and +2.7. Considering that the oxidation state of Co correlates linearly with the K‐edge energy derived from the first derivative of the absorption spectrum, a calibration line based on Co foil and Co_3_O_4_ standards was established [38, 39]. The fitted result indicated that the average valence state of cobalt in Co‐SAC was approximately +1.9 (Figure S3), implying partial oxidation of the Co centers coordinated within the nitrogen‐doped carbon matrix. Wavelet transform (WT) analysis further differentiated Co‐SAC from metallic cobalt, displaying a distinct single intensity maximum centered at ∼4 Å^−1^ and 1.4 Å, which can be unequivocally assigned to Co–N coordination rather than Co–Co scattering (Figure 1g; Figures S4 and S5). Similarly, the Fourier‐transformed EXAFS spectra in the R‐space exhibit only one prominent peak at approximately 1.4 Å (Figure 1h,i), corresponding to the Co–N shell and confirming the atomic dispersion of Co without any detectable Co–Co contributions. Quantitative EXAFS curve fitting yields a coordination number of ∼4.1, consistent with a well‐defined Co–N_4_ configuration (Figure 1i,j).

Collectively, these spectroscopic results unambiguously verify the successful formation of atomically isolated cobalt sites with Co–N_4_ coordination geometry, which provides abundant and well‐exposed active centers favorable for PMS activation and catalytic oxidation processes.

### 2.2 Catalytic Performance of CoN_4_

The catalytic activity of CoN_4_ toward PMS activation was systematically investigated using SMX as a typical contaminant, as detailed in Text S4. In the absence of PMS, CoN_4_ (50 mg/L) partially removed SMX (∼20% removal in 30 min), likely via adsorption onto the hierarchically etched hollow structure of the catalyst with a large specific surface area. In the system containing only PMS, ∼10% SMX was removed within 30 min, while the introduction of the catalyst led to complete removal (100%) within 5 min (Figure S6). Notably, the observed pseudo‐first‐order SMX degradation rate constant (*k*_obs_) for the PMS/CoN_4_ system (i.e., 0.736 min^−1^) is 67‐fold higher than that for the PMS‐only system (0.011 min^−1^), underscoring the essential role of Co single‐atom sites in PMS activation.

**FIGURE 2:**
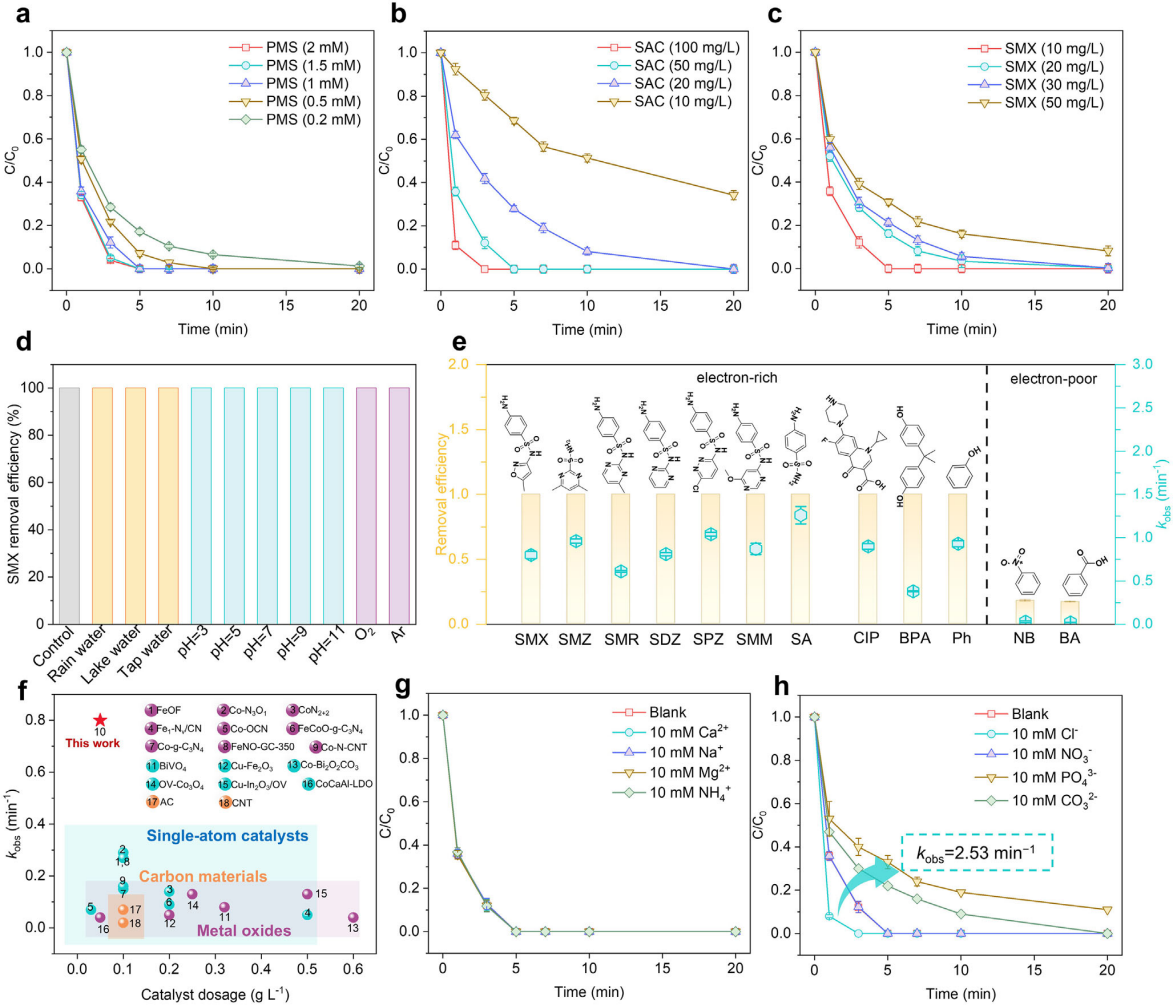
Catalytic performance of CoN_4_. SMX degradation in different (a) PMS concentrations, (b) CoN_4_ dosages, and (c) SMX concentrations in PMS/CoN_4_ systems (Conditions: PMS = 1 mm, CoN_4_ = 50 mg L^−^^1^, SMX = 10 mg L^−^^1^). (d) Effects of water matrices, pH, and atmosphere on SMX degradation in the PMS/CoN_4_ system. (e) Degradation efficiencies of different ECs. (f) Comparison of the *k*_obs_ of pollutants degradation with catalysts reported in the literature, see catalyst, oxidant, contaminant, and the corresponding dosages in Table S4. Effects of cations (g) and anions (h) on SMX degradation.

To gain deeper insight into the SMX degradation performance of the PMS/CoN_4_ system, a series of controlled experiments was conducted. The catalyst was added prior to each degradation test, and PMS was introduced only after an equilibration period of ∼30 min (denoted as *t* = 0). The effects of PMS concentration, catalyst dosage, and initial SMX concentration on degradation performance were systematically investigated in the PMS/CoN_4_ system (Figure 2a–c). Increasing the catalyst dosage (10–100 mg L^−1^) or PMS concentration (0.2–2.0 mm) markedly enhanced SMX degradation, reflecting the dependence of the reaction rate on active site density and oxidant availability. Conversely, higher initial SMX concentrations (10–50 mg L^−1^) resulted in slower degradation kinetics due to the increased pollutant load. To evaluate the practical applicability of the PMS/CoN_4_ system, the effects of the water matrices, pH, and atmospheric conditions were systematically investigated. SMX underwent almost complete degradation within 15 min in various water matrices of rain, lake, and tap water (Figure 2d; Figure S7, Table S2), demonstrating the high reactivity of the PMS/CoN_4_ system across different water environments. It should be noted that the degradation kinetics decreased with increasing COD_Mn_ in real water matrices. The modest inhibition observed is likely due to the competitive quenching of reactive species by coexisting natural organic matter (NOM) and/or dissolved organic matter present in these samples [40, 41]. Furthermore, variations in the pH (3–11) and atmospheric conditions (O_2_ and Ar) exerted only minor influences (Figure 2d; Figures S8 and S9). Collectively, these results highlight the robustness and practical applicability of the PMS/CoN_4_ system. The degradation performance of the PMS/CoN_4_ system was subsequently assessed for a broad spectrum of refractory ECs. For electron‐rich compounds, including SMX, sulfamethazine (SMZ), sulfamerazine (SMR), sulfadiazine (SDZ), sulfachloropyridazine (SPZ), sulfamonomethoxine (SMM), sulfanilamide (SA), ciprofloxacin (CIP), bisphenol A (BPA), and phenol (Ph), removal efficiencies exceeding 90% were achieved within 7 min (Figure 2e; Figure S10), highlighting the excellent catalytic performance of PMS/CoN_4_ system. In contrast, the removal efficiencies for electron‐poor compounds, such as nitrobenzene (NB) and benzoic acid (BA), were below 20%, indicating the preferential degradation of electron‐rich contaminants. Furthermore, the PMS/CoN_4_ system was compared with several state‐of‐the‐art catalytic systems for the degradation of emerging organic pollutants (Figure 2f; Table S4). This analysis, which specifically focused on catalyst dosage, oxidant concentration, and kinetic rates, revealed that the PMS/CoN_4_ system demonstrated rapid reaction kinetics (0.736 min^−1^) with low catalyst dosage and low oxidant concentration, surpassing the performances of previously reported SACs, metal oxides, and carbon‐based systems (Figure 2f).

Inorganic ions are ubiquitous in natural and engineered water matrices and are known to modulate the reactivity, speciation, and transformation pathways of reactive oxidants, thereby influencing pollutant degradation kinetics [33, 34]. To assess the robustness of the PMS/CoN_4_ system toward such matrix effects, the influence of common cations and anions on sulfamethoxazole (SMX, 10 mg L^−1^) degradation under standard conditions was systematically examined under standard conditions (PMS = 1 mm, CoN_4_ = 50 mg L^−1^, Figure 2g,h). Upon the individual introduction of representative cations (Ca^2^^+^, Na^+^, Mg^2^^+^, or NH_4_^+^, 10 mm) or anions (Cl^−^, NO_3_^−^, PO_4_^3^^−^, or CO_3_^2^^−^, 10 mm), the SMX removal efficiency at 20 min remained close to 100% in all cases, underscoring the excellent stability and interference resistance of the PMS/CoN_4_ system under ion‐rich conditions. The presence of PO_4_^3^^−^ and CO_3_^2^^−^ led to only a minor decrease of the apparent rate, which can be ascribed to weak complexation or precipitation involving surface metal sites [42]; however, this effect did not compromise the near‐complete pollutant removal. Strikingly, Cl^−^ displayed a markedly distinct behavior. As the Cl^−^ concentration increased from 0 to 10 mm, the observed *k*_obs_ for SMX degradation rose markedly from 0.736 to 2.53 min^−1^ (Figure 2h; Figure S11), indicating a pronounced acceleration effect induced by Cl^−^ addition. The results demonstrate that Cl^−^ promotes SMX degradation within the tested range, though the kinetic acceleration is significantly lower than that observed at higher concentrations. Specifically, as the chloride concentration increased from 0 to 2 mm, *k*_obs_ rose from 0.736 to 1.068 min^−1^. This suggests that while the promotional effect of chloride is concentration‐dependent, it remains observable even under more realistic, low‐chloride conditions. It is important to consider that while low Cl^−^ levels are typical for many effluents, high‐salinity scenarios are also common in practice. For instance, certain pharmaceutical and industrial wastewaters can contain chloride levels of 1–5 g/L, which far exceed the millimolar range focused on in this study [43]. Recycling experiments demonstrated that the CoN_4_ catalyst maintained excellent stability irrespective of the presence of chloride ions (Figure S12 and Table S5). After 10 consecutive cycles, both the PMS/CoN_4_ and PMS/Cl^−^/CoN_4_ systems consistently achieved high SMX removal efficiencies. Inductively coupled plasma mass spectrometry (ICP‐MS) analysis of the supernatants collected after each cycle revealed cobalt concentrations consistently below 0.015 mg L^−^^1^ (Figure S13a), indicating only trace‐level leaching. Control experiments showed that the equivalent concentration of Co^2+^ in a Co^2+^/PMS system exhibited no obvious degradation of SMX (Figure S13b), thereby excluding any significant homogeneous contribution to the catalytic performance. Compared with the fresh catalyst, the XRD pattern and microstructure of the used CoN_4_ showed no discernible changes (Figure S14a,b), confirming that Co atoms remained atomically dispersed after 10 cycles. Although a slight decline in degradation efficiency was observed over successive cycles, the CoN_4_ catalyst remains highly competitive relative to many recently reported single‐atom systems for pollutant degradation (Table S6). Such mild deactivation is commonly ascribed to unavoidable catalyst loss during centrifugation and recovery, rather than the degradation of the active‐site structure [44].

### 2.3 Reaction Mechanisms of PMS/CoN_4_

**FIGURE 3:**
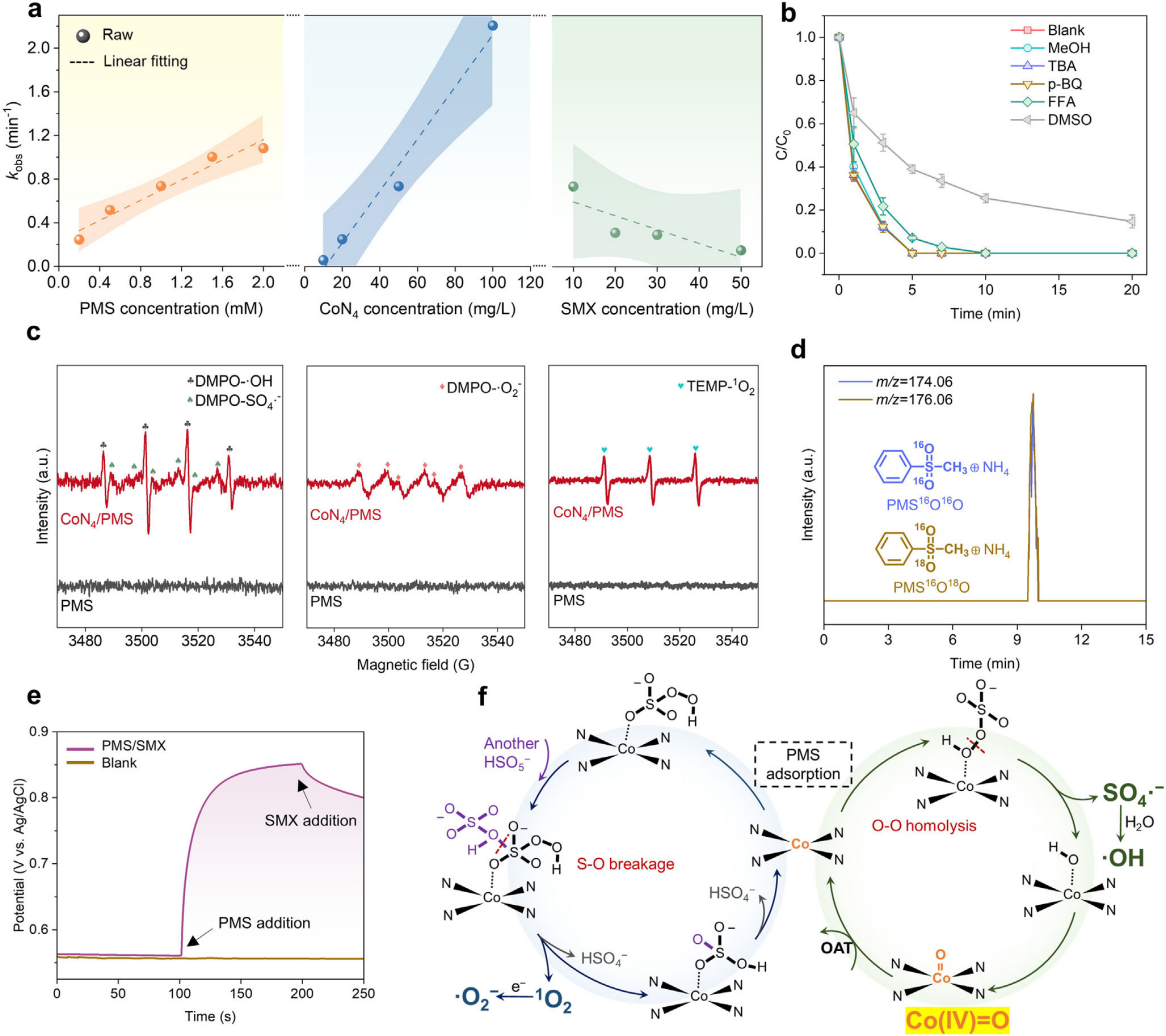
Reaction mechanism of the PMS/CoN_4_ system. (a) Effects of PMS concentration, CoN_4_ dosage, and SMX concentration on *k*_obs_. (b) Quenching experiments with various scavengers. (c) EPR spectra of the PMS/CoN_4_ system with DMPO and TEMP. (d) EIC of PMS^16^O^16^O and PMS^16^O^18^O generated during PMS^16^O oxidation in the PMS/CoN_4_ system in H_2_^18^O. (e) OCP curves on the CoN_4_ electrode. (f) Schematic of the radical and non‐radical synergistic formation for CoN_4_.

To clarify the factors controlling the reaction rate in the PMS/CoN_4_ system, the variation in *k*_obs_ with PMS, CoN_4_, and SMX contents was evaluated (Figure 3a; Figures S15–S17). Notably, *k*_obs_ increased linearly with increasing PMS and CoN_4_ contents, but decreased as the initial SMX concentration was increased. These trends indicate that the amounts of catalyst and oxidant primarily govern SMX degradation, whereas direct electron transfer involving the pollutant plays only a minor role [45]. In metal SAC based advanced oxidation processes, multiple ROS can form, including hydroxyl and sulfate radicals (^•^OH and SO_4_^•−^), superoxide (^•^O_2_^−^), singlet oxygen (^1^O_2_), and high‐valent metal–oxo species. To determine which species are responsible for SMX removal in the PMS/CoN_4_ system, quenching experiments were performed (Table S7). Methanol (MeOH) was used to quench both ^•^OH and SO_4_^•−^, while *tert*‐butanol (TBA) targeted ^•^OH [46]. As shown in Figure 3b and Figure S18, inhibiting ^•^OH and SO_4_^•−^ led to only a slight decrease in SMX degradation. Similar behavior was observed when *p*‐benzoquinone (*p*‐BQ), a scavenger of ^•^O_2_^−^, was added. These results indicate that radical species contribute to SMX oxidation but are not the dominant pathway. Considering non‐radical oxidation pathways, the introduction of furfuryl alcohol (FFA), a quencher of ^1^O_2_, led to a noticeable decrease in the SMX degradation rate, whereas the addition of dimethyl sulfoxide (DMSO), a scavenger of high‐valent metal species, caused an even more pronounced suppression of SMX removal [47, 48]. These results show that both singlet oxygen and high‐valent metal species are key oxidants in the PMS/CoN_4_ system, with the high‐valent metal pathway playing the larger role. This suggests that the nitrogen coordination environment around the Co atoms promotes their interaction with PMS and favors non‐radical oxidation, consistent with previous reports on Co‐based SACs [22].

To further verify the identities and roles of the reactive species in the PMS/CoN_4_ system, EPR analyses were performed using different spin‐trapping agents. Using 5,5‐dimethyl‐1‐pyrroline *N*‐oxide (DMPO), characteristic signals were detected corresponding to DMPO–·OH, DMPO–SO_4_^•−^, and DMPO–^•^O_2_^−^ adducts, confirming the formation of these radicals, despite their minor contribution to SMX degradation. In contrast, the addition of 2,2,6,6‐tetramethylpiperidine (TEMP) produced a clear 1:1:1 triplet signal corresponding to the TEMP–^1^O_2_ adduct (Figure 3c), confirming the generation of singlet oxygen [49]. Notably, in control systems containing PMS alone, no radicals or singlet oxygen species were detected. Additionally, the involvement of high‐valent cobalt–oxo species was probed via an oxygen atom transfer pathway using H_2_^18^O as the reaction medium [50]. In the presence of labeled water, both PMS^16^O^16^O (m/z 174.06 [M+NH_4_]^+^) and PMS^16^O^18^O (m/z 176.06 [M+NH_4_]^+^) were detected (Figure 3d), indicating that oxygen from water was incorporated into PMS, and supporting the formation of Co(IV) = O. Moreover, to quantitatively address the roles of all detectable reactive species suggested by the EPR spectra, the oxidative contributions of individual ROS were further deconvoluted using the quenching‐based approach (Text S10) [51, 52] and summarized in Figure S19. The results reveal a pronounced dominance of non‐radical oxidants, with Co(IV) = O and ^1^O_2_ accounting for 77.44% and 17.67% of SMX abatement, respectively. In contrast, radical pathways contributed only marginally, with ^•^OH, SO_4_^•−^, and ^•^O_2_^−^ responsible for merely 0.82%, 2.72%, and 1.09% of SMX removal, respectively, while the remaining 0.26% was attributed to other minor routes. These quantitative data corroborate that, although multiple ROS are generated, the PMS/CoN_4_ system predominantly operates through a Co(IV) = O‐centered non‐radical regime, with ^1^O_2_ serving as a secondary oxidant, whereas free radicals play a negligible role in governing the overall degradation kinetics.

Direct electron transfer (DET) is often proposed in PMS‐based catalytic systems; therefore, its role in the PMS/CoN_4_ system was investigated using electrochemical measurements. The open‐circuit potential (OCP) was recorded in a single‐chamber cell with CoN_4_ as the working electrode (Text S12). After PMS addition, the OCP increased rapidly by ∼ 0.30 V (Figure 3e), indicating the formation of strong surface oxidants, such as Co(IV) = O. The subsequent introduction of SMX caused a slight decrease in the OCP, suggesting that PMS‐derived species (PMS^*^) may participate in DET between the pollutant and the catalyst surface. The minor OCP change suggests that the contribution of DET is likely negligible, consistent with the ROS contribution analysis.

As summarized in Figure 3f, PMS activation by CoN_4_ proceeds through both free‐radical and non‐radical pathways. The radical route involves one‐electron transfer to generate sulfate and hydroxyl radicals, whereas the non‐radical route involves two‐electron transfer to form singlet oxygen and a high‐valent cobalt–oxo intermediate. These pathways operate in parallel and drive SMX oxidation in the PMS/CoN_4_ system. Consistent with this dual‐pathway mechanism, radical quenching experiments showed that ^•^OH, SO_4_^•−^ and ^•^O_2_^−^ only partially inhibited SMX degradation, indicating a major contribution from non‐radical species. Moreover, EPR measurements detected signals from both radical species and ^1^O_2_, confirming their simultaneous formation. Overall, the results identify Co(IV) = O as the primary oxidizing species responsible for pollutant removal. Moreover, the observation that Cl^−^ markedly enhances SMX degradation raises the possibility that Cl^−^ promotes pollutant removal by facilitating the formation of Co(IV) = O. Mechanistic experiments were therefore conducted to evaluate this possibility.

### 2.4 Chloride‐Enhanced Co(IV) = O Formation

Chloride is ubiquitous in natural and engineered waters and has long been recognized as a key matrix constituent that can reshape PMS‐based oxidation chemistry. In radical‐mediated PMS systems, Cl^−^ is often considered a “transforming/quenching” ion because it readily reacts with primary radicals (e.g., SO_4_^•^^−^ and ^•^OH), diverting them into reactive chlorine species (RCS) such as chlorine radicals (Cl^•^/Cl_2_^•^^−^) and free chlorine (HOCl/ClO^−^), which typically exhibit distinct selectivity and oxidative capacity compared with the parent radicals [53].

**FIGURE 4:**
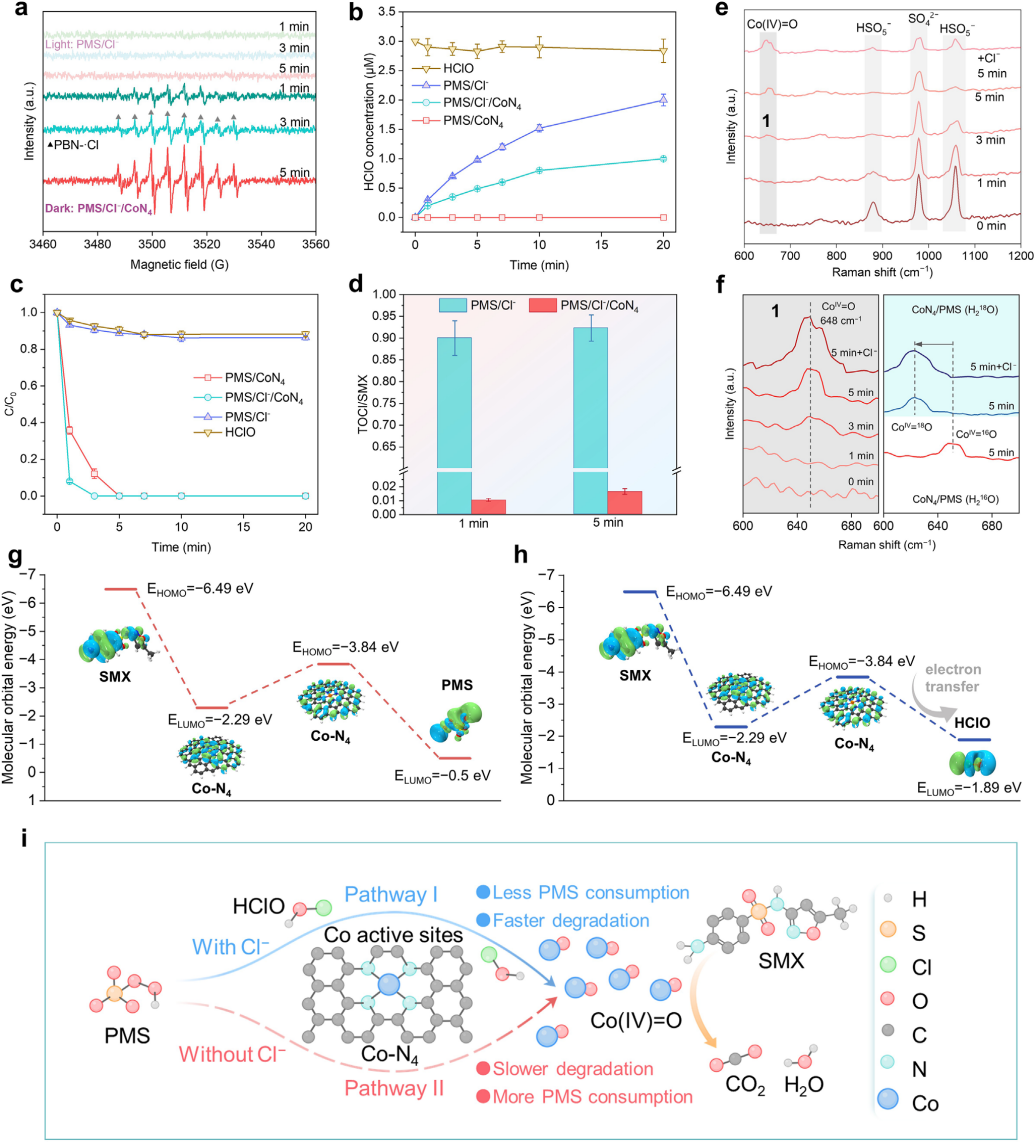
Reaction mechanism of the Cl‐added system. (a) EPR signal intensity of PBN‐Cl^•^ in PMS/Cl^−^ (light lines) and PMS/Cl^−^/CoN_4_ (dark lines) systems over time. (b) Variations in the concentration of HClO as a function of reaction time in different systems. (c) Variations in SMX removal by different systems. (d) Ratio of total organochlorine formation after the degradation of per unit concentration of SMX in various systems. Experimental conditions: [SMX] = 10 mg L^−^^1^, CoN_4_ = 50 mg L^−^^1^, [PMS] = 1 mm, [Cl^−^] = 10 mm, [HClO] = 3 µm for (b–d). (e) In situ time‐dependent SERS of CoN_4_ in a PMS‐Cl^−^ system. (f) Enlarged view labeled 1 in (e) and in situ SERS of CoN_4_ in a PMS‐Cl^−^ system obtained in H_2_^16^O/H_2_^18^O matrix. (g–h) The molecular orbital energy diagram of SMX, PMS, HClO, and CoN_4_. (i) Mechanism diagram of dual pathways for Co(IV) = O generation.

To examine whether RCS were responsible for the accelerated SMX degradation observed in the PMS/Cl^−^/CoN_4_ system, EPR experiments were conducted to probe chlorine‐radical‐related signals. As shown in Figure 4a, no discernible chlorine radical signal was detected in the PMS/Cl^−^ system, whereas characteristic radical features emerged distinctly when CoN_4_ was introduced (PMS/Cl^−^/CoN_4_), implying that CoN_4_ facilitates the conversion of PMS‐derived oxidizing equivalents into a chlorine‐involved reactive pool. Meanwhile, the formation of HOCl was directly quantified (Text S9; Figure S20). As illustrated in Figure 4b, the concentration of HOCl in the HOCl‐only control remained essentially unchanged over 0–20 min (initially ∼3 µm), confirming its stability under the testing conditions. In contrast, HOCl accumulated progressively from 0 to ∼2 µm in the PMS/Cl^−^ system within 20 min, supporting that PMS activation in the presence of chloride can generate free chlorine. This is consistent with the well‐established pathway in sulfate‐radical chemistry, where SO_4_^•^^−^ oxidizes Cl^−^ to Cl^•^ (SO_4_^•^^−^ + Cl^−^ → SO_4_^2^^−^ + Cl^•^), followed by secondary reactions yielding Cl_2_^•^^−^ and, ultimately, HOCl/ClO^−^ in water [54]. Notably, the HOCl level in PMS/Cl^−^/CoN_4_ increased initially but plateaued at a lower value (∼1 µm at 10 min) compared with PMS/Cl^−^ alone, indicating that CoN_4_ actively consumes the in situ generated HOCl rather than simply promoting its accumulation. This observation aligns with the proposed chloride‐enabled catalytic route, in which HOCl serves as a reactive intermediate that couples to the Co‐centered redox cycle rather than acting as the terminal oxidant.

The reactivity tests further exclude HOCl and chlorine radicals as the dominant driver for SMX abatement. As shown in Figure 4c, neither HOCl alone nor PMS/Cl^−^ exhibited meaningful oxidative removal of SMX, with only ∼10% degradation after 20 min, demonstrating that HOCl itself cannot account for the ultrafast degradation kinetics observed in PMS/Cl^−^/CoN_4_. Importantly, in many chloride‐containing AOPs, accelerated degradation is often accompanied by enhanced formation of toxic chlorinated organic byproducts, reflected by increased TOCl [55, 56, 57]. However, TOCl analysis revealed a stark contrast (Figure 4d): oxidation of unit SMX in PMS/Cl^−^/CoN_4_ generated negligible TOCl compared with PMS/Cl^−^, strongly suggesting that rapid SMX depletion in the catalytic system does not proceed through indiscriminate chlorination by RCS, but instead follows a more selective non‐radical oxidative route.

In situ surface‐enhanced Raman spectroscopy (SERS) was employed to monitor the evolution of high‐valent cobalt species in real time. As shown in Figure 4e, PMS displayed the characteristic peaks of HSO_5_^−^ at around 889 and 1063 cm^−^^1^, along with the peak of SO_4_^2^^−^ at 984 cm^−^^1^ [58, 59, 60]. Notably, the PMS/CoN_4_ system developed a distinct band at 648 cm^−^^1^ over 1–5 min, attributable to the formation of Co(IV) = O [61]. Strikingly, upon addition of Cl^−^ at 5 min, the 648 cm^−^^1^ signal intensified immediately, indicating that chloride introduction substantially promotes Co(IV) = O accumulation. Since Co(IV) = O species can undergo oxygen atom exchange (OAE) with H_2_O, the identification of ^18^O‐labeled products in H_2_^18^O media provides compelling evidence for its existence. Upon performing the reaction in an H_2_^18^O matrix, the characteristic Co(IV) = O stretching vibration at 648 cm^−^^1^ exhibited a discernible redshift (Figure 4f). This isotopic shift, originating from the replacement of ^16^O by ^18^O, aligns well with established literature [62]. Moreover, the HOCl/CoN_4_ system also exhibited the same 648 cm^−^^1^ feature (Figure S19), providing direct evidence that HOCl can effectively drive the formation of Co(IV) = O on CoN_4_. Collectively, these results demonstrate that Cl^−^ enhances SMX degradation not by RCS‐dominated oxidation, but by generating a transient HOCl intermediate that accelerates the Co‐centered high‐valent pathway, thereby amplifying the Co(IV) = O population responsible for the ultrafast, selective oxidation.

To gain a molecular‐level insight into the mechanism by which Cl^−^ ions enhance Co(IV) = O formation, DFT calculations were performed (Figure 4g,h). In the PMS/CoN_4_ system, Cl^−^ promotes the in situ formation of HOCl due to the reaction between Cl^−^ and SO_4_^•−^. Frontier orbital analysis showed that HOCl (LUMO = −1.89 eV) interacts more readily with CoN_4_ than with SMX. Additionally, the HOMO of CoN_4_ (−3.84 eV) is significantly higher in energy than the HOMO of SMX (−6.49 eV), leading to a smaller energy gap between the HOMO of CoN_4_ and the LUMO of HOCl (∼1.95 eV) compared to that between the HOMO of SMX and the LUMO of HOCl (∼4.60 eV). The reduced energy gap suggests stronger frontier orbital interactions, implying a more thermodynamically favorable electron‐transfer from CoN_4_ to HOCl. Consequently, electrons are preferentially transferred from CoN_4_ to HOCl, enabling HOCl to oxidize the Co center to Co(IV) = O via a thermodynamically favorable low‐barrier pathway. Thus, the catalyst serves as an electron‐accepting bridge, channeling the oxidizing power of HOCl toward high‐valent Co formation rather than nonspecific pollutant chlorination. Overall, the alignment of HOCl (LUMO = −1.89 eV), CoN_4_ (HOMO = −3.84 eV; LUMO = −2.29 eV), and SMX (HOMO = −6.49 eV) defines a lower‐energy pathway for Co(IV) = O production in the presence of Cl^−^ ions. These theoretical results are fully consistent with the experimental data, confirming that Cl^−^ accelerates the formation of Co(IV) = O and improves the catalytic selectivity of the PMS/CoN_4_ system.

Specifically, in the Cl^−^‐free PMS/CoN_4_ system, Co(IV) = O accounted for ∼77.4% of the overall oxidative contribution, while at higher Cl^−^ concentrations, both the quenching results and the calculated contribution ratios showed a progressive increase in the proportion of Co(IV) = O‐mediated oxidation (Figure S22). In Cl^−^‐containing systems, the addition of DMSO, a selective scavenger for high‐valent metal species, immediately suppressed SMX degradation, confirming the reinforcing role of Co(IV) = O. Importantly, the oxidant contributions estimated from quenching analysis reflect the relative distribution among parallel oxidation pathways rather than the absolute formation flux or steady‐state concentration of each oxidant [63]. Therefore, the marked increase in *k*_obs_ upon Cl^−^ addition, despite only a slight increase in the relative Co(IV) = O contribution, indicates that chloride mainly enhances the absolute generation/regeneration rate of Co(IV) = O while Co(IV) = O remains the dominant oxidant. This interpretation is consistent with the immediate enhancement of the 648 cm^−^^1^ Raman signal assigned to Co(IV) = O after Cl^−^ addition and with the observation that HOCl/CoN_4_ also produces the same Co(IV) = O feature. When the Cl^−^ concentration reached 100 mM, the contribution of high‐valent cobalt species increased to nearly 90%. PMS decomposition over time, both in the presence and absence of Cl^−^ was also compared. In the Cl^−^‐free system, PMS was consumed rapidly, with ∼20% decomposition being observed within 20 min (Figure S23). In contrast, PMS decayed significantly more slowly in the presence of Cl^−^, with a larger fraction of PMS remaining at each time point (e.g., ∼88% remaining after 20 min). This indicates that lower PMS doses achieve pollutant removal comparable to the Cl^−^‐free system. In other words, Cl^−^ promotes the selective use of PMS by favoring Co(IV) = O formation over unproductive radical pathways and associated oxidant consumption. This behavior is consistent with recent reports on Fe‐based PMS systems, in which Cl^−^ addition increased pollutant degradation rates while significantly reducing PMS consumption [64]. Therefore, the slower PMS depletion observed here supports the conclusion that CoN_4_ and Cl^−^ act synergistically to favor a non‐radical pathway dominated by Co(IV) = O while minimizing excess PMS decomposition.

Figure 4i conceptually summarizes the two routes to Co(IV) = O formation in the CoN_4_/PMS system and highlights the mechanism by which Cl^−^ introduces an additional pathway. Specifically, in the absence of Cl^−^ ions (Pathway I), PMS directly oxidizes the CoN_4_ active site to generate a high‐valent Co(IV) = O species, which then oxidizes SMX and drives pollutant degradation. This direct PMS oxidation route operates in both Cl^−^‐free and Cl^−^‐containing systems and represents the baseline non‐radical pathway. In the presence of Cl^−^ ions (Pathway II), the second route becomes dominant. Initially, PMS oxidizes Cl^−^ to form HOCl. Rather than HOCl reacting directly with or chlorinating SMX, it is intercepted by the CoN_4_ catalyst. HOCl rapidly oxidizes the Co sites to generate Co(IV) = O, releasing Cl^−^ back into solution. The Co(IV) = O produced in this manner oxidizes SMX in the same manner as in Pathway I. Thus, Cl^−^ effectively acts as a cocatalyst, converting PMS into the key Co(IV) = O intermediate via HOCl, thereby reinforcing the non‐radical oxidation pathway.

### 2.5 Evaluation of Practical Applications

**FIGURE 5:**
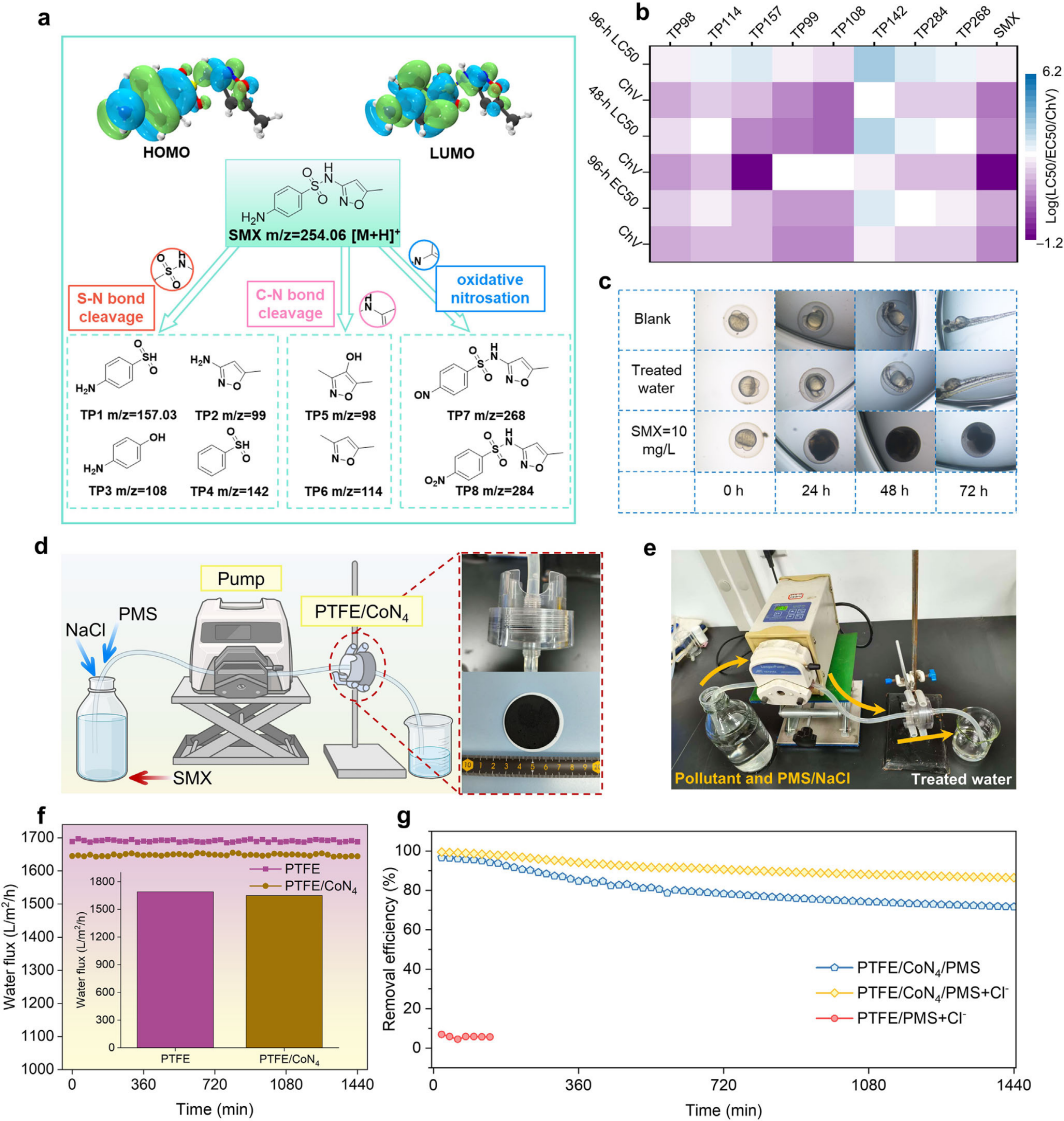
Environmental risk assessment, continuous‐flow catalytic performance, and operational durability of CoN_4_. (a) Proposed degradation pathways for the SMX degradation. (b) Ecotoxicity assessment of SMX and its transformation products toward fish, daphnid, and green algae. (c) Images of zebrafish embryos under different treatments: nutrient medium only (Blank), post‐reaction SMX solution from the PMS/CoN_4_ system, and SMX solution. (d) Schematic illustration of the wastewater treatment process, scale‐up part shows the membrane reactor and a PTFE‐supported CoN_4_ membrane. (e) Photograph of the wastewater treatment experimental equipment. (f) Water flux during the continuous‐flow process with and without CoN_4_‐loaded PTFE membranes. Insets show the averaged water flux values. (g) SMX removal efficiency of different systems.

Understanding the reactive sites of SMX is essential for clarifying its degradation behavior in the PMS/CoN_4_ system. DFT calculations were performed to predict the most vulnerable positions on the SMX molecule. As shown in Figure 5a and Figure S24, the HOMO is mainly localized on the N11 atom and the aniline ring, indicating that electron‐rich regions are likely targets for oxidation. The electrostatic potential map further shows negatively charged areas (blue) concentrated around O and N atoms and in the π‐electron region (Figure S25), suggesting that these sites are prone to electron‐withdrawing reactions by strong oxidants such as Co(IV) = O and ^1^O_2_.

To provide supplementary spectroscopic evidence for SMX consumption during the reaction, UV–vis spectra before and after degradation were recorded after quenching with methanol. As shown in Figure S26, the characteristic absorption band of SMX at around 260 nm was prominent before reaction but gradually weakened and eventually disappeared as the reaction proceeded, qualitatively indicating the depletion of the parent SMX molecule [65].

While UV–vis spectroscopy provides evidence for SMX disappearance, high‐performance liquid chromatography–mass spectrometry (HPLC–MS) is more informative for resolving transformation intermediates and elucidating the degradation process at the molecular level. Transformation products (TPs) identification before and after the degradation (Figures S27 and S28) presented three possible degradation pathways of SMX. In Pathway I, the non‐radical oxidative cleavage of the sulfonamide S─N bond yields TP1 (*m/z* 157.03), TP2 (*m/z* 99, undetected), TP3 (*m/z* 108), and TP4 (*m/z* 142). In Pathway II, cleavage of the C─N bond generates TP5 (*m/z* 98, undetected) and TP6 (*m/z* 114), while Pathway III involves an electrophilic attack on the aniline moiety, generating TP7 (*m/z* 268) and TP8 (*m/z* 284). These routes are consistent with the calculated reactive sites and observed TPs, indicating that bond cleavage and modification around the sulfonamide and aniline segments dominate SMX degradation. The environmental relevance of these transformations was assessed by estimating the aquatic toxicity of SMX and its TPs using Ecological Structure–Activity Relationships (ECOSAR) [66, 67, 68]. The predicted toxicity differed among species, with daphnid being the most sensitive, followed by green algae and then fish, in line with Globally Harmonized System ‐based hazard classifications. The majority of TPs exhibited lower predicted toxicities than the parent SMX, indicating an overall reduction in ecotoxicity during treatment (Figure 5b; Table S8). Beyond the computational insights, the biosafety of the treated water was further assessed using a zebrafish embryo development assay. Exposure to the SMX solution induced pronounced acute toxicity, manifested by early mortality and abnormal swimming behavior within 24 h. By contrast, zebrafish maintained in the treated effluent collected after the PMS/CoN_4_ reaction displayed normal viability and behavior with no detectable adverse effects (Figure 5c), underscoring the biocompatibility of the purified water. Collectively, these observations support the environmental compatibility of the PMS/CoN_4_ process for efficient SMX abatement.

Building on the mechanistic understanding and high catalytic efficiency of the PMS/CoN_4_ system, its practicality was further assessed at the device level. By coupling advanced oxidation with polytetrafluoroethylene (PTFE) membrane confinement, we developed a catalytic filtration platform that suppresses pollutant dispersion while enabling self‐cleaning operation. The membrane was prepared by vacuum‐assisted deposition of 100 mg CoN_4_ suspension onto PTFE supports, yielding a flexible and mechanically robust catalytic layer suitable for continuous‐flow wastewater treatment (Figure 5d–e). The hydraulic behavior of the pristine PTFE and PTFE/CoN_4_ membranes was benchmarked by analyzing the mass transfer and permeation of SMX‐containing solutions (Figure 5f). Notably, the functionalized membrane sustained stable performance over 24 h, delivering a water flux of 1650.1 L m^−^^2^ h^−^^1^, comparable to that of bare PTFE (1690.7 L m^−^^2^ h^−^^1^), indicating minimal flow resistance after catalyst integration. The simulated SMX wastewater (10 mg L^−^^1^) was treated both in the presence and absence of Cl^−^ ions (0 or 10 mm). Notably, in the presence of Cl^−^, SMX removal remained >85% throughout the 24 h operation (Figure 5g), consistent with the Cl^−^‐promoted formation of Co(IV) = O described above. In both chloride‐containing and chloride‐free systems, the leached cobalt concentration (2 µg L^−^^1^) was far below the Chinese standard for surface water sources used for drinking water supply (1 mg L^−^^1^) (Figure S29). However, in the absence of CoN_4_ loading, the pristine PTFE membrane reactor exhibited negligible SMX removal, indicating that catalytic activity is essential for effective degradation. Overall, these results suggest that Cl^−^ acts as a practical kinetic lever for enhancing Co(IV) = O‐mediated, non‐radical SMX removal, and support the feasibility of implementing the PMS/CoN_4_ system in a membrane‐based continuous‐flow format with verified biosafety. Although 10 mm Cl^−^ does not represent all wastewater scenarios, the continuous‐flow comparison under chloride‐free and chloride‐containing conditions demonstrates that the Cl^−^‐enhanced Co(IV) = O pathway can be effectively translated from mechanistic batch tests to device‐level operation, particularly for chloride‐containing or saline water matrices.

## 3 Conclusion

In this work, an atomically dispersed CoN_4_ SAC was synthesized via a template‐assisted pyrolysis–etching route. The SAC exhibited exceptional PMS‐activation performance for the degradation of SMX (*k*_obs_ = 0.736 min^−1^). Specifically, the PMS/CoN_4_ system completely removed SMX within minutes and maintained strong resistance against coexisting ions, pH fluctuations, and diverse water matrices. Notably, the presence of Cl^−^ ions led to enhanced SMX degradation, yielding a degradation rate constant of 2.53 min^−1^. Mechanistic investigations revealed that Co(IV) = O contributes up to 77.4% of the total oxidizing power of the system, establishing it as the principal reactive species. In situ Raman and DFT calculations showed that Cl^−^ ions provide an energetically favorable pathway for Co(IV) = O formation, in which in situ‐generated HOCl facilitates electron transfer from CoN_4_ and substantially lowers the activation barrier for Co─O bond oxidation. This Cl^−^‐mediated route not only enhanced the formation of Co(IV) = O, but also suppressed non‐productive PMS decomposition, thereby improving catalytic selectivity and promoting oxidant utilization. Moreover, a CoN_4_‐loaded PTFE membrane reactor sustained >85% SMX removal during continuous operation in 24 h, demonstrating excellent stability under realistic water conditions and facilitating its application in practical scenarios. Collectively, this study elucidates a previously unrecognized Cl^−^‐promoted high‐valent cobalt pathway and provides mechanistic guidance for designing SAC‐driven PMS systems for selective, efficient, and sustainable water treatment.

## 4 Experimental Section

Experimental details are provided in the Supporting Information.

### 4.1 Statistical Analysis

All catalytic degradation experiments were independently conducted in triplicate (*n* = 3) unless otherwise specified, and the data are presented as mean ± standard deviation (SD).

## Conflicts of Interest

The authors declare no conflicts of interest.

## Supporting information




**Supporting File**: advs75549‐sup‐0001‐SuppMat.docx.

## Data Availability

The data that supports the findings of this study are available in the supplementary material of this article.
