# Supplementary material for: Cobalt Single‐Atom Catalysts for Ultrafast Sulfamethoxazole Degradation: Unveiling the Chloride‐Ion‐Enhanced Formation of Co(IV)=O
Source: Adv Sci (Weinh). 2026 May 5;13(42):e75549. doi: 10.1002/advs.75549 (PMC13336067; doi:10.1002/advs.75549)
Supplement: Supplementary file 1 — Supporting File: advs75549‐sup‐0001‐SuppMat.docx. [file ADVS-13-e75549-s001.docx]

**Cobalt Single-Atom Catalysts for Ultrafast Sulfamethoxazole Degradation: Unveiling the Chloride-Ion-Enhanced Formation of Co(IV)=O**

Anting Ding^1^, Jing Lu^2^, Yafei Fan^3^, Chenyu Zeng^1^, Yuhang Lin^1^, Yufei Shi^1^, Zulin Zhang^1,4^

^1^Xianghu Laboratory, Hangzhou 311231, China

^2^Department of Environmental Engineering, College of Environmental & Resource Sciences, Zhejiang University, Hangzhou, 310058, China

^3^Key Lab for colloid and Interface Science of Ministry of Education, School of Chemical and Chemical Engineering, Shandong University, Jinan, 250100, China

^4^The James Hutton Institute, Craigiebuckler, Aberdeen AB15 8QH, UK

**Correspondence:**

Yufei Shi (Email:shiyufeix@163.com) Zulin Zhang (E-mail: zulin.zhang@hutton.ac.uk)

**Keywords:** single-atom catalysts, peroxymonosulfate, high-valent cobalt, chloride ions, sulfamethoxazole

Anting Ding, Jing Lu and Yafei Fan contributed equally to this work.

# CONTENTS

**Texts.**

Text S1. Chemicals and Materials.

Text S2. Synthesis of CoN_4_.

Text S3. Materials characterization.

Text S4. Catalytic activity evaluation.

Text S5. Reusability and recycling tests.

Text S6. Detection of target pollutants.

Text S7. Total organic chlorine (TOCl) analysis.

Text S8. PMS concentration determination.

Text S9. HClO concentration determination.

Text S10. ROS analysis.

Text S11. ^18^O isotope tracing experiments.

Text S12. Electrochemical measurements.

Text S13. Computational Methods.

Text S14. Detection of degradation intermediates.

Text S15 Zebrafish embryo toxicity assessment.

**Figures.**

Figure S1. Nitrogen adsorption-desorption isotherms and pore size distribution of Co-SACs.

Figure S2. Raman spectra and PXRD pattern of Co-SACs.

Figure S3. Analysis spectra of the Co valence state from the corresponding Co K-edge.

Figure S4. Wavelet transform analysis of Co K-edge EXAFS data of Co_3_O_4_ and Co foil.

Figure S5. EXAFS analysis of Co foil and Co_3_O_4_ in k and R space.

Figure S6. SMX degradation/adsorption performance at different conditions.

Figure S7. Degradation of SMX in real water matrices.

Figure S8. Degradation of SMX under different pH.

Figure S9. Influence of different aeration conditions on SMX degradation.

Figure S10. Degradation of various organic pollutants in the CoN_4_/PMS system.

Figure S11. SMX degradation in the PMS/Cl^−^/CoN_4_ system with varied Cl^−^ concentration.

Figure S12. Cyclic SMX removal in PMS/CoN_4_ and PMS/Cl^−^/CoN_4_ systems.

Figure S13. Cobalt leaching in 10 cycles and SMX degradation in homogeneous systems.

Figure S14. XRD pattern of the catalysts before and after cycles and HAADF-STEM image of the used catalysts.

Figure S15. SMX degradation kinetics with different PMS concentrations.

Figure S16. SMX degradation kinetics with different CoN_4_ dosages.

Figure S17. SMX degradation kinetics with different SMX concentrations.

Figure S18. Quenching experiments with various scavengers and the corresponding *k*_obs_.

Figure S19. Contribution of different ROS for SMX degradation.

Figure S20. Corresponding relationship between the concentration and absorbance of HClO.

Figure S21. Raman spectra of CoN_4_/HClO system.

Figure S22. Co(IV)=O contribution ratio in PMS/Cl^−^/CoN_4_ with varied Cl^−^ concentration.

Figure S23. PMS consumption rates in PMS/Cl^−^/CoN_4_ with varied Cl^−^ concentration.

Figure S24. Optimized molecular structure of SMX.

Figure S25. The ESP of the SMX molecule.

Figure S26. UV-vis spectra of SMX during degradation.

Figure S27. Total ion chromatogram and MS spectra of the reaction solution before degradation.

Figure S28. Total ion chromatogram and MS spectra of the reaction solution after SMX degradation.

Figure S29. Co leaching efficiency using the SACs-loaded PTFE membrane.

**Tables.**

Table S1. EXAFS fitting structural parameters of CoN_4_ at the Co K-edge.

Table S2. Characteristics of real aqueous medium.

Table S3. HPLC parameters for the analysis of different pollutants.

Table S4. Cyclic SMX degradation ratio and removal efficiency in PMS/CoN_4_ and PMS/Cl^−^/CoN_4_ systems.

Table S5. Comparison of catalytic performance of different catalysts in PMS-based systems.

Table S6. Comparison of cycling durability of recent single-atom catalysts for pollutant degradation.

Table S7. Second-order reaction rate constants between different organics and ROS (M⁻^1^ s⁻^1^).

Table S8. Ecotoxicity assessment of SMX and its transformation products towards fish, daphnid and green algae.

**References.**

Ref. [1]−[36]

# TEXT

**Text S1.** Chemicals and Materials.

Analytical standard chemicals and HPLC-grade solvents were mainly obtained from Shanghai Macklin Biochemical Co., Ltd. The purchased reagents included peroxymonosulfate (PMS), sulfamethoxazole (SMX), sulfamethazine (SMZ), sulfamerazine (SMR), sulfadiazine (SDZ), sulfachloropyridazine sodium (SPZ), sulfamonomethoxine (SMM), sulfanilamide (SA), ciprofloxacin (CIP), bisphenol A (BPA), phenol (Ph), nitrobenzene (NB), benzoic acid (BA), furfuryl alcohol (FFA), tertiary butanol (TBA), cobaltous chloride tetrahydrate. Additional chemicals, including methanol (MeOH), dimethyl sulfoxide (DMSO), sodium dihydrogen phosphate, sodium carbonate, sodium sulfate, sodium thiosulfate, calcium nitrate, sulfuric acid, sodium hydroxide, and potassium iodide (KI), were purchased from Sinopharm Chemical Reagent Co., Ltd. 5,5-Dimethyl-1-pyrrolidine N-oxide (DMPO) and 2,2,6,6-tetramethylpiperidine (TEMP) were obtained from Dojindo Laboratories. Phenylmethyl sulfoxide (PMSO), phenylmethyl sulfone (PMSO_2_), oxygen-18-labeled water (H_2_^18^O), and p-benzoquinone (p-BQ) were supplied by Aladdin Biological Technology Co., Ltd. All aqueous solutions were prepared using ultrapure water (resistivity >18 MΩ·cm) obtained from a Milli-Q purification system.

**Text S2.** Synthesis of CoN_4_.

1 g of SiO_2_ was dispersed in 100 mL of ethanol, followed by the addition of 1 g of o-phenylenediamine and 0.418 g of CoCl_2_·4H_2_O. The mixture was vigorously stirred at room temperature for 12 h and then subjected to rotary evaporation. The resulting solid was heated under an argon atmosphere to 800 °C at a ramping rate of 5 °C min^−1^ and maintained for 2 h to induce carbonization. Subsequently, the product was treated with 2.0 M NaOH and H_2_SO_4_ solutions to remove the SiO_2_ template and residual Co nanoparticles. After thorough washing with deionized water and drying in a vacuum oven at 70 °C for 12 h, the CoN_4_ catalyst was obtained.

**Text S3.** Materials characterization.

The surface morphologies of the materials were characterized using field-emission scanning electron microscopy (FESEM, Hitachi SU8010). Their internal structures were further examined by transmission electron microscopy (TEM, JEOL JEM-2100F, 200 kV). High-angle annular dark-field scanning transmission electron microscopy (HAADF-STEM, FEI Spectra 300, 300 kV) was employed to visualize the atomically dispersed Co sites. X-ray diffraction (XRD) patterns were obtained on a ZETIUM DY 2186 diffractometer equipped with a Cu Kα radiation source. The specific surface areas and N₂ adsorption–desorption isotherms were determined using a Micromeritics ASAP 2460 analyzer based on the Brunauer–Emmett–Teller (BET) method. X-ray photoelectron spectroscopy (XPS) analyses were conducted on a Thermo Scientific K-Alpha instrument with an Al Kα X-ray source. Metal ion concentrations were quantified by inductively coupled plasma optical emission spectroscopy (ICP-OES, iCAP PRO, Thermo Fisher Scientific, USA). Electron paramagnetic resonance (EPR) measurements were performed on a Bruker EMX PLUS spectrometer (Germany) under the following conditions: center field 3510 G, sweep width 100 G, static field 3460 G, microwave power 6.325 mW, microwave frequency 9.85 GHz, modulation frequency 100 kHz, modulation amplitude 1.0 G, and sweep time 30 s. X-ray absorption fine structure (XAFS) spectra were recorded at the BL14W1 beamline of the Shanghai Synchrotron Radiation Facility (SSRF, China) using a Si(111) double-crystal monochromator, with a storage ring energy of 3.5 GeV and an operating current of 260 mA. The incident X-ray intensity was monitored by a nitrogen-filled ionization chamber, and all spectra were collected in transmission mode with a beam size of approximately 0.3 mm × 0.3 mm at the sample position. All measurements were carried out at room temperature. Raman spectra were measured using a Raman spectroscopy instrument (Horiba Scientific) equipped with a laser emitting at 532 nm.

**Text S4.** Catalytic activity evaluation.

Experiments were conducted in 100 mL centrifuge tubes, each containing approximately 100 mL of reaction solution composed of PMS (1 mM), the target pollutant (10 mg·L^−1^), and CoN_4_ (50 mg·L^−1^). The tubes were mounted on a magnetic stirrer and agitated at 600 rpm in the dark at 25 ± 0.5 °C. At selected time points, 0.5 mL samples were withdrawn, immediately mixed with 0.5 mL methanol and 50 mM sodium thiosulfate to quench the reaction, passed through 22 μm polytetrafluoroethylene (PTFE) filters, and then analyzed without delay. Each condition was tested in triplicate, and the error bars shown in the figures correspond to the standard deviations of parallel experiments.

**Text S5.** Reusability and recycling tests.

Catalyst stability was evaluated through consecutive cycling experiments for both the PMS/CoN_4_ and PMS/Cl⁻/CoN_4_ systems. After each degradation run, the CoN_4_ catalyst was recovered by centrifugation and filtration, thoroughly rinsed with deionized water, and dried prior to reuse in the subsequent cycle. All experimental parameters were kept constant throughout the five cycles (PMS = 1 mM, CoN_4_ = 50 mg L⁻^1^, SMX = 10 mg L⁻^1^; Cl⁻ = 10 mM when applicable). For each cycle, the SMX removal efficiency was quantified at 5 min for the PMS/CoN_4_ system and at 1 min for the PMS/Cl⁻/CoN_4_ system. The degradation performance in the first cycle was normalized to 100%, and the relative degradation efficiency for each subsequent cycle was calculated by dividing the removal percentage of that cycle by the corresponding value in the first cycle.

**Text S6.** Detection of target pollutants.

The target pollutants were quantified using high-performance liquid chromatography (HPLC, ACQUITY UPLC, Agilent) fitted with a TC-C18 column (150 × 4.6 mm). Analyses were carried out under isocratic conditions, with a mobile phase composed of 0.1% acetic acid aqueous solution and acetonitrile. The flow rate was set to 0.8 mL min^−1^, the injection volume was 10 μL, and the column temperature was maintained at 30 °C. The detailed detection wavelengths and corresponding mobile phase compositions are provided in Table S3.

**Text S7.** Total organic chlorine (TOCl) analysis.

The total organic chlorine (TOCl) concentration was detected as follows: 100 mL reaction solution (filtered through 0.22 μm water phase film) was enriched on an activated carbon column using pretreatment equipment (APU-2, analytikjena) and then determined by microcoulometric method using a total organic halogen analyzer (MultiX 2500, analytikjena). The ratio of total organochlorine formation after the degradation of per unit concentration of SMX in various systems was calculated by dividing the measured total TOCl yield by the total amount of SMX degraded during the reaction.

**Text S8.** PMS concentration determination.

The PMS concentration was measured using a UV spectrophotometric approach. Briefly, 0.5 mL of the reaction solution was added to 9.5 mL of a reagent solution containing 1 g KI and 0.1 g NaHCO_3_. After a reaction time of 15 min, the absorbance at 352 nm was recorded on a UV–Vis spectrophotometer (Thermo Evolution 201).

**Text S9.** HClO concentration determination.

Active chlorine concentration was quantified via a DPD (N, N-diethyl-1,4-phenylenediamine) colorimetric method at 515 nm. In the presence of PMS, excess EDTA-Na_2_ was added to quench PMS interference. Specifically, 0.5 mL phosphate buffer, 0.5 mL DPD, 2.0 mL EDTA-Na_2_ solution, and 1.0 mL ultrapure water were mixed, followed by the addition of 1.0 mL reaction solution. The absorbance was recorded immediately at 515 nm^[1]^. This setup ensured selective measurement of HClO in systems containing both PMS and active chlorine. Calibration curves showed strong linearity (R^2^ > 0.99), confirming the reliability of this method.

**Text S10.** ROS analysis.

The degradation behavior of contaminants in the catalyst/PMS system was fitted using a pseudo-first-order kinetic model. The apparent reaction rate constant (*k_obs_*) was obtained based on Eq. (S1):

$\mathrm{In}\left( \frac{C_{t}}{C_{0}} \right)=-k_{obs} \times t$ (S1)

where *C_t_* and *C_0_* denote the contaminant concentrations at time *t* and the initial time, respectively.

To clarify the roles of various ROS in the degradation process, a series of quenching agents (i.e., TBA, MeOH, DMSO, *p*-BQ, and FFA) were employed to selectively scavenge the corresponding reactive species. The second-order reaction rate constants between these quenching agents and different ROS are summarized in Table S6. The contribution ratios of different ROS were calculated according to Eqs. (S2)−(S6).

$R_{\cdot OH}=(k_{control}-k_{TBA})/k_{control}$ (S2)

$R_{\mathrm{SO}_{4}^{\cdot-}}=(k_{TBA}-k_{MeOH})/k_{control}$ (S3)

$R_{1_{O2}}=(k_{MeOH}-k_{FFA})/k_{control}$ (S4)

$R_{O_{2}^{\cdot-}}=(k_{MeOH}-k_{p-BQ})/k_{control}$ (S5)

$R_{\mathrm{Co}\left( \mathrm{IV} \right)=O}=(k_{MeOH}-k_{DMSO})/k_{control}$ (S6)

Where R represents the contribution ratio of each ROS, *k_control_* is the *k_obs_* in the absence of scavengers, and *k_scavenger_* denotes the rate constant obtained in the presence of the corresponding scavenger (e.g., TBA, MeOH, FFA, p-BQ).

**Text S11.** ^18^O isotope tracing experiments

Isotope-labeling experiments were conducted with H_2_^18^O as the oxygen donor. Typically, 1 mL of H_2_^18^O solution containing 3 mM PMSO was first prepared, after which PMS (1 mM) and CoN_4_ (50 mg L⁻^1^) were introduced. Following a reaction time of 3 min, the mixture was collected and subjected to LC–MS analysis to detect the generation of ^16^O/^18^O-labeled PMSO_2_ and related species.

**Text S12.** Electrochemical measurements.

To fabricate the working electrode, 10 mg of catalyst was dispersed in a solvent mixture of 0.9 mL ethanol, 0.1 mL deionized water, and 50 μL Nafion solution (5 wt%). The suspension was ultrasonicated for 30 min to obtain a uniform catalyst ink, after which 200 μL of the ink was drop-cast onto a 1 cm² piece of carbon paper and dried. Electrochemical tests were carried out using both an H-type electrolytic cell and a conventional three-electrode setup. In the H-type cell, the two compartments were separated by a proton exchange membrane and each was filled with 50 mM Na_2_SO_4_ electrolyte. The chamber containing SMX served as the working electrode side, whereas the PMS-containing chamber was used for the counter and reference electrodes. For the three-electrode system, the catalyst-loaded carbon paper, a platinum foil, and an Ag/AgCl electrode acted as the working, counter, and reference electrodes, respectively. Open-circuit potential (OCP) measurements were performed under this configuration.

**Text S13.** Computational Methods.

ORCA (revision 6.0.1)^[2, 3]^ was utilized for Density Functional Theory (DFT) calculations. Structure optimizations were carried out at the PBE0/def2-SVP level of theory^[4, 5]^, augmented by the D3 dispersion correction with Becke-Johnson (BJ) damping^[6]^ to accurately model long-range van der Waals interactions. Following these optimizations, which yielded structures free of imaginary frequencies, single point energies and frontier orbitals were subsequently calculated at the PBE0/def2-TZVP level. The analysis of these frontier orbitals was performed using Multiwfn (revision 2.8(dev))^[7, 8]^. Visual Molecular Dynamics (VMD, revision 1.9.3)^[9]^ facilitated the generation of images for the optimized structures and frontier orbitals.

**Text S14** Detection of degradation intermediates.

The oxidation intermediates of SMX were analyzed by high-performance liquid chromatography-mass spectrometry (HPLC-MS, Agilent 6460, USA). A Zorbax SB-C18 column (3.5 μm, 2.1 × 150 mm) was used for separation under full scan in positive ion mode with a scanning range of m/z 30–300. The column temperature was maintained at 30°C, and the flow rate was 0.3 mL·min⁻¹. The mobile phase A was 0.05% formic acid solution, and mobile phase B was methanol. A gradient program was applied as follows: 0-2 min, 5% B; 2-17 min, 5-95% B; 17-20 min, 95% B. The injection volume was 10 μL. The drying gas temperature and flow rate were set at 325°C and 5 L·min^-^¹, respectively. The nebulizer pressure was 45 psi, the sheath gas temperature was 350 °C with a flow rate of 11 L·min^-^¹, and the capillary voltage was 3000 V.

**Text S15** Zebrafish embryo toxicity assessment.

Three healthy adult zebrafish (one female and two males) were co-housed in a spawning tank containing nutrient medium for 12 h to induce spawning and maximize embryo yield. Fertilized embryos were collected, thoroughly rinsed with deionized water to remove surface contaminants, and screened under a stereomicroscope. Embryos at the eight-cell stage were selected for subsequent toxicity assays. Exposure solutions were prepared according to the experimental design. Briefly, embryos in the treatment group were exposed to the post-reaction solution collected from the PMS/CoN_4_ catalytic system (SMX-containing water after reaction). The blank group was maintained in nutrient medium only, while the control group was exposed to an untreated SMX solution. Embryos were distributed into 24-well culture plates (one embryo per well, ten embryos per group). Each well contained 1 mL of nutrient medium mixed with 1 mL of the designated exposure solution. Embryo development was monitored microscopically, and the exposure solutions were renewed every 24 h from the start of treatment until hatching. oxidation intermediates of SMX were analyzed by high-performance liquid chromatography-mass spectrometry.

# FIGURE


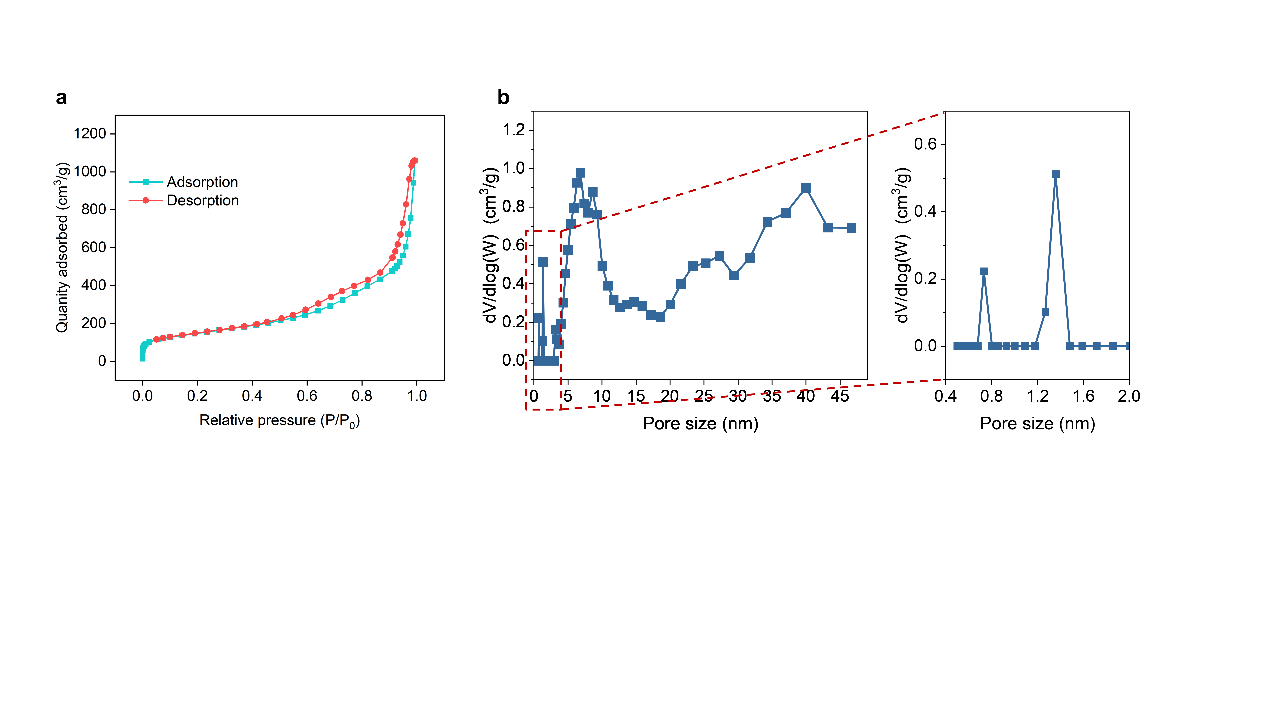


**Figure S1.** (a) Nitrogen adsorption-desorption isotherms of Co-SACs. (b) Pore size distribution of Co-SACs.


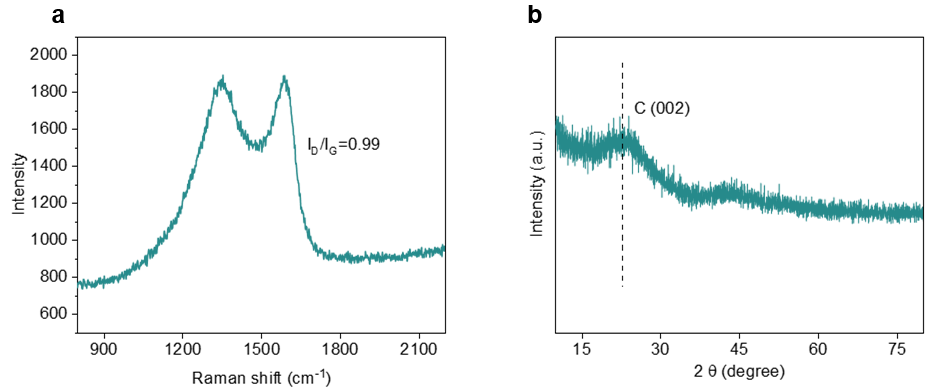


**Figure S2.** (a) Raman spectrum and (b) PXRD pattern of Co-SACs.





**Figure S3.** Analysis spectra of the Co valence state from the corresponding Co K-edge.


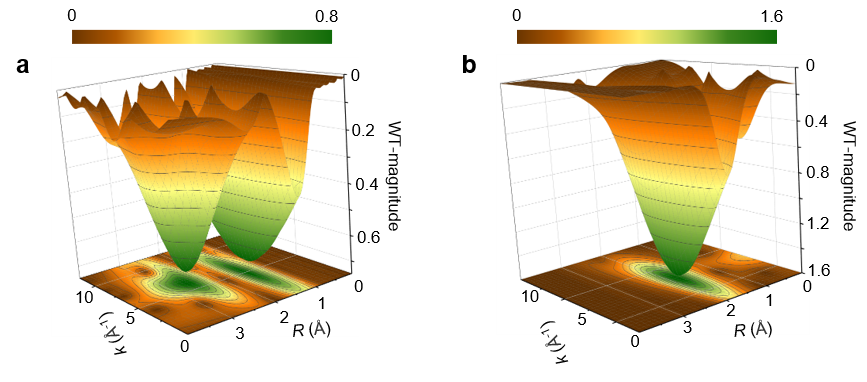


**Figure S4.** Wavelet transform analysis of Co K-edge EXAFS data of Co_3_O_4_ (a) and Co foil (b).


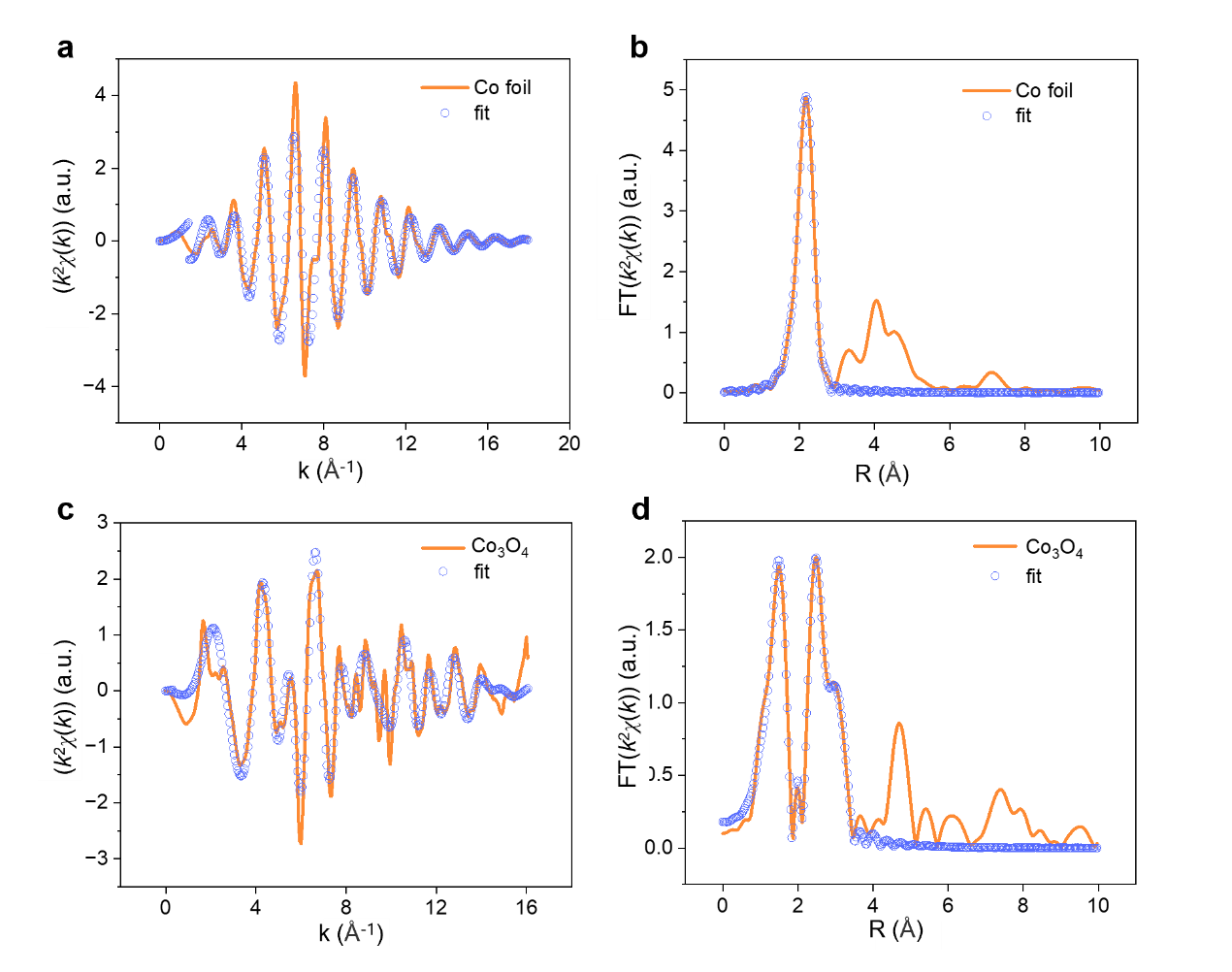


**Figure S5.** The EXAFS analysis of Co foil in k (a) and R (b) space; the EXAFS analysis of Co_3_O_4_ in k (c) and R (d) space.





**Figure S6.** SMX degradation/adsorption performance at different conditions.





**Figure S7.** Degradation of SMX in real water matrices.





**Figure S8.** Degradation of SMX under different pH.





**Figure S9.** Influence of different aeration conditions on SMX degradation.





**Figure S10.** Degradation of various organic pollutants in the CoN_4_/PMS system.

**
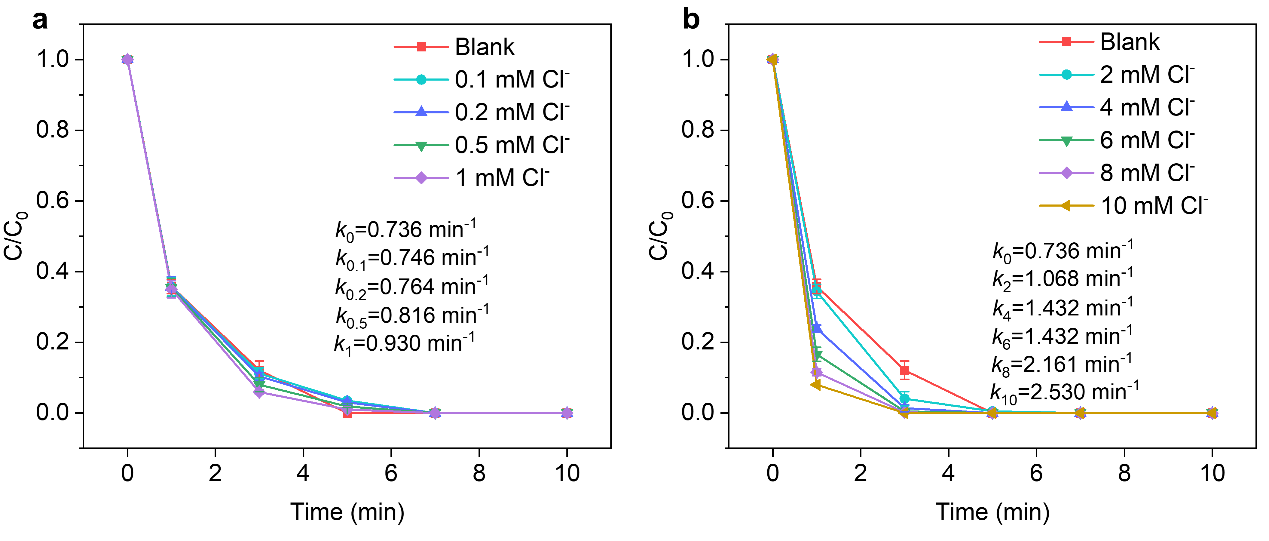
**

**Figure S11.** Degradation of SMX in the PMS/Cl^−^/CoN_4_ system with varied Cl^−^ concentration of (a) 0−1 mM and (b) 0−10 mM.


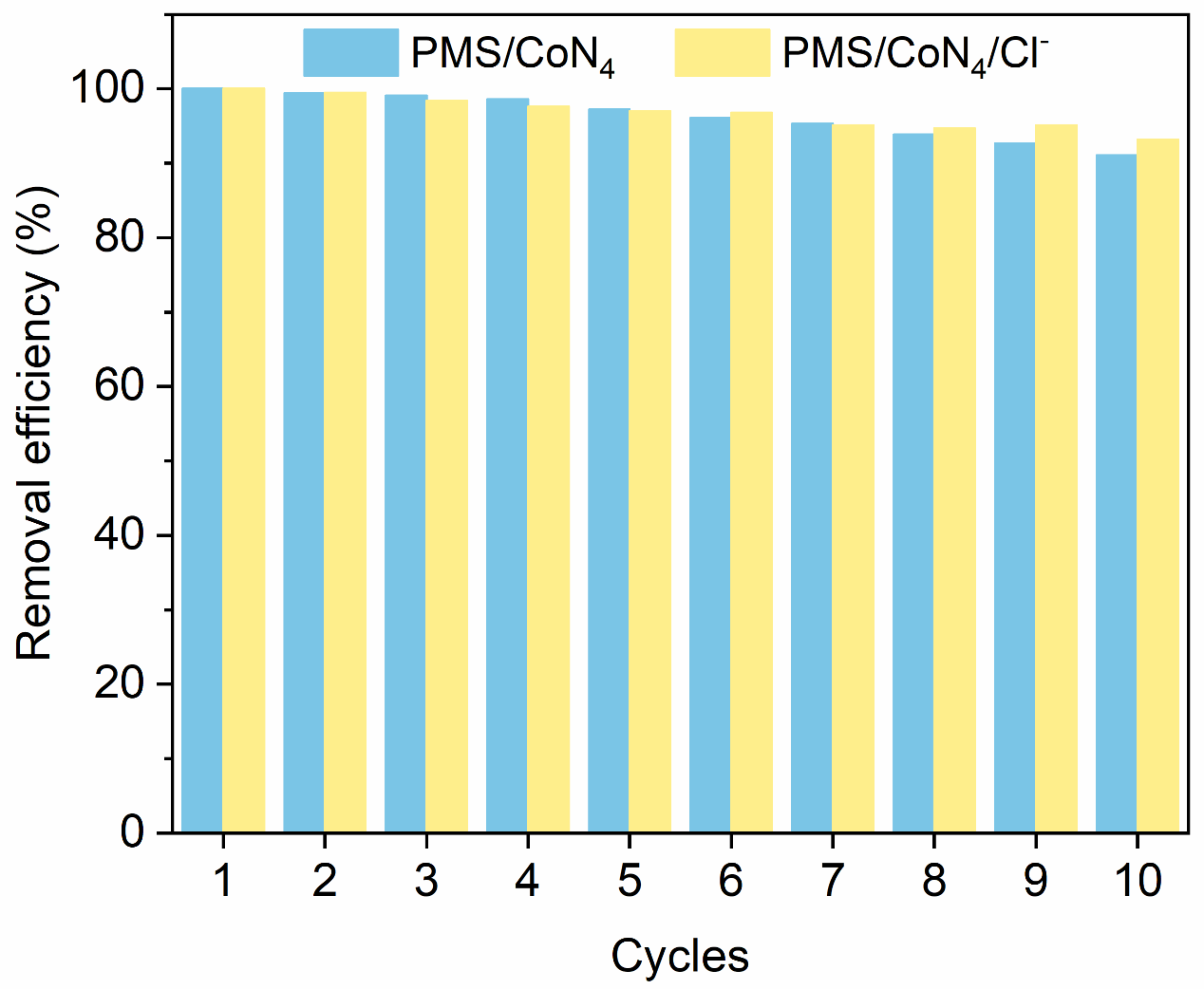


**Figure S12.** Cylic SMX removal efficiency in CoN_4_/PMS and CoN_4_/PMS/Cl^−^ systems.


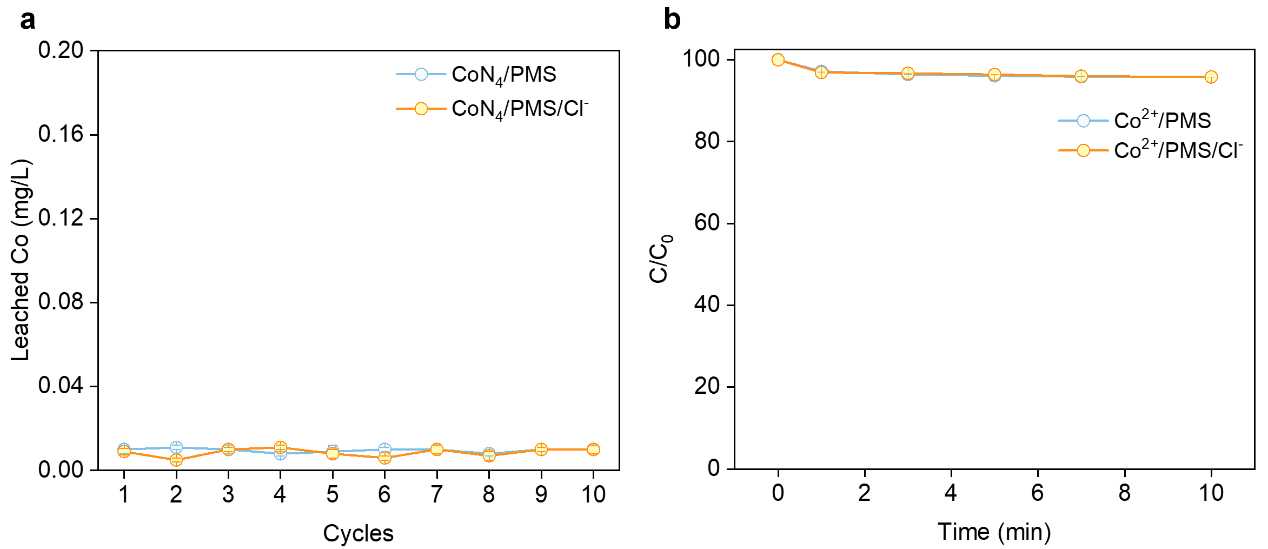


**Figure S13.** (a) Cobalt leaching in CoN_4_/PMS and CoN_4_/PMS/Cl^−^ systems in 10 cycles. (b) SMX degradation in Co^2+^/PMS and Co^2+^/PMS/Cl^−^ systems (Conditions: PMS = 1 mM, Co^2+^ = 0.015 mg L⁻^1^, SMX= 10 mg L⁻^1^).


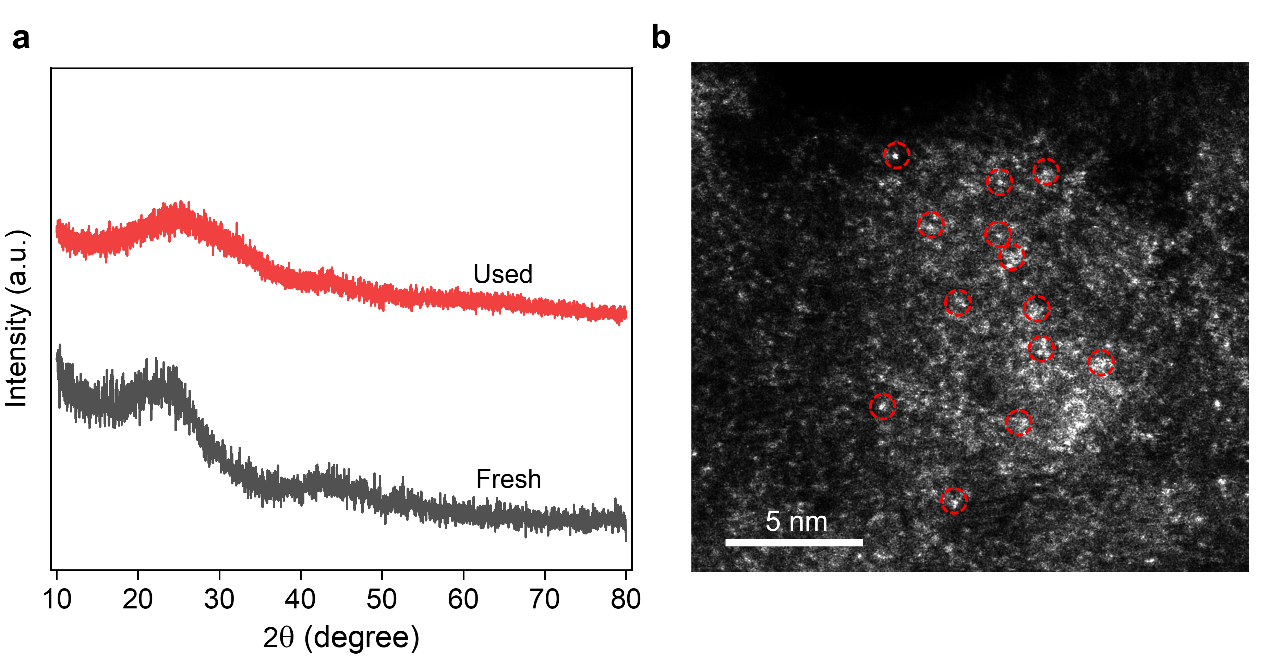


**Figure S14.** (a) The XRD pattern of Co-SACs before and after 10 cycles in CoN_4_/PMS/Cl^−^ system. (b) HAADF-STEM image of the used Co-SACs after 10 cycles in CoN_4_/PMS/Cl^−^ system, where the single atoms are marked with red circles.





**Figure S15.** SMX degradation kinetics with different PMS concentrations.





**Figure S16.** SMX degradation kinetics with different CoN_4_ dosages.





**Figure S17.** SMX degradation kinetics with different SMX concentrations.


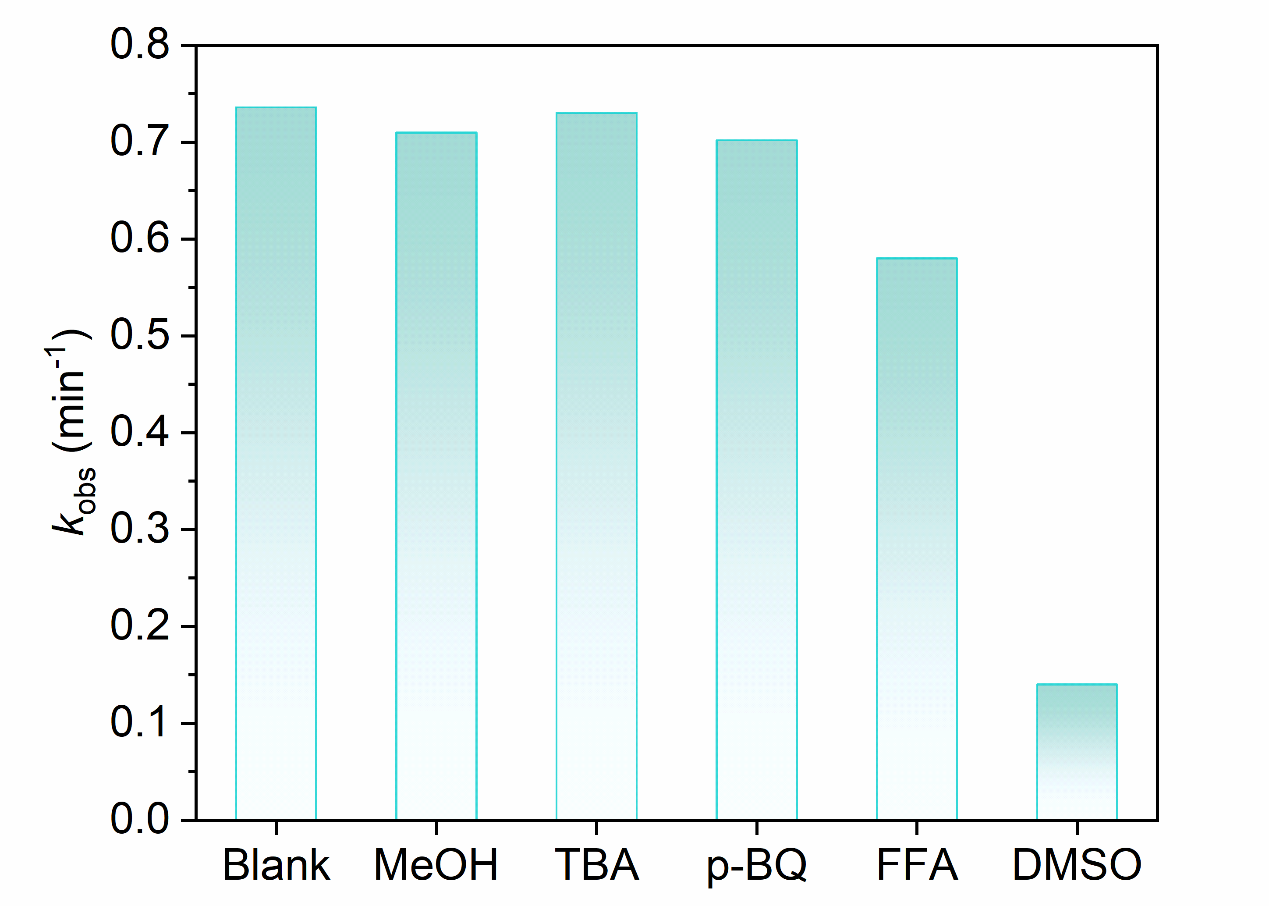


**Figure S18.** Quenching experiments with various scavengers and the corresponding *k*_obs_.


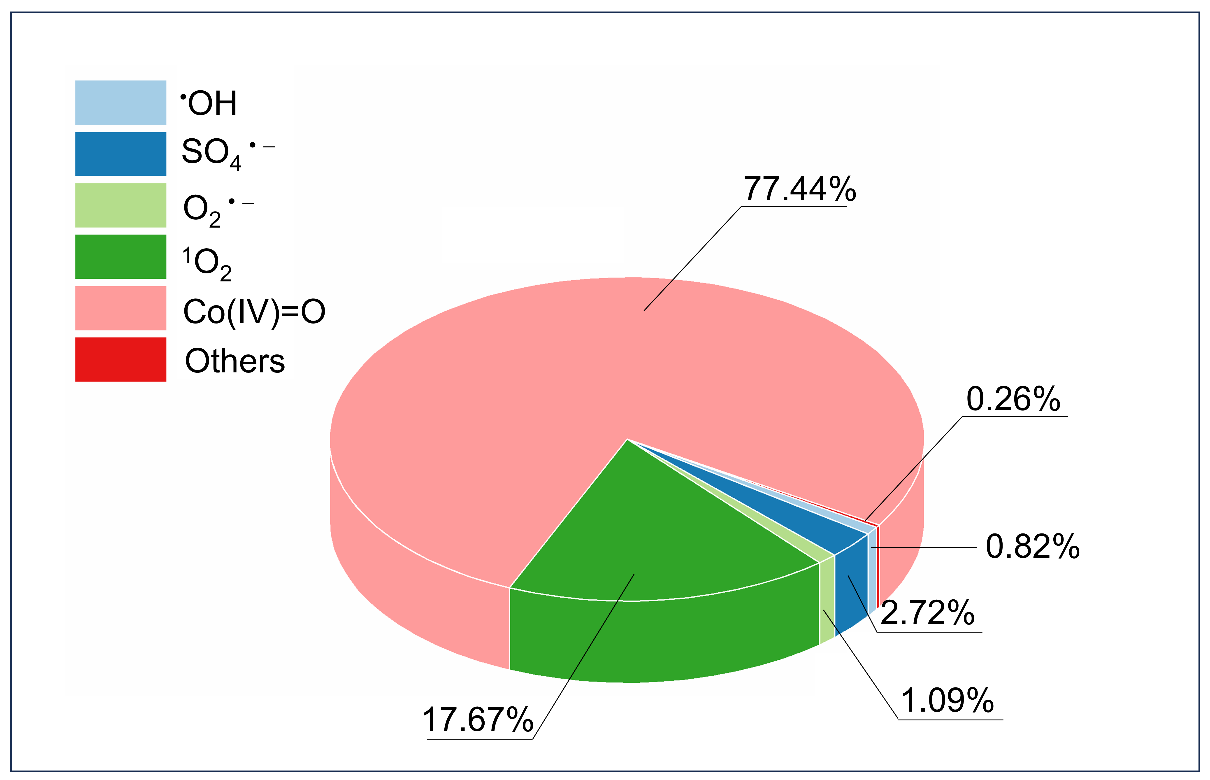


**Figure S19.** Contribution of different ROS for SMX degradation.





**Figure S20.** Corresponding relationship between the concentration and absorbance of HClO.


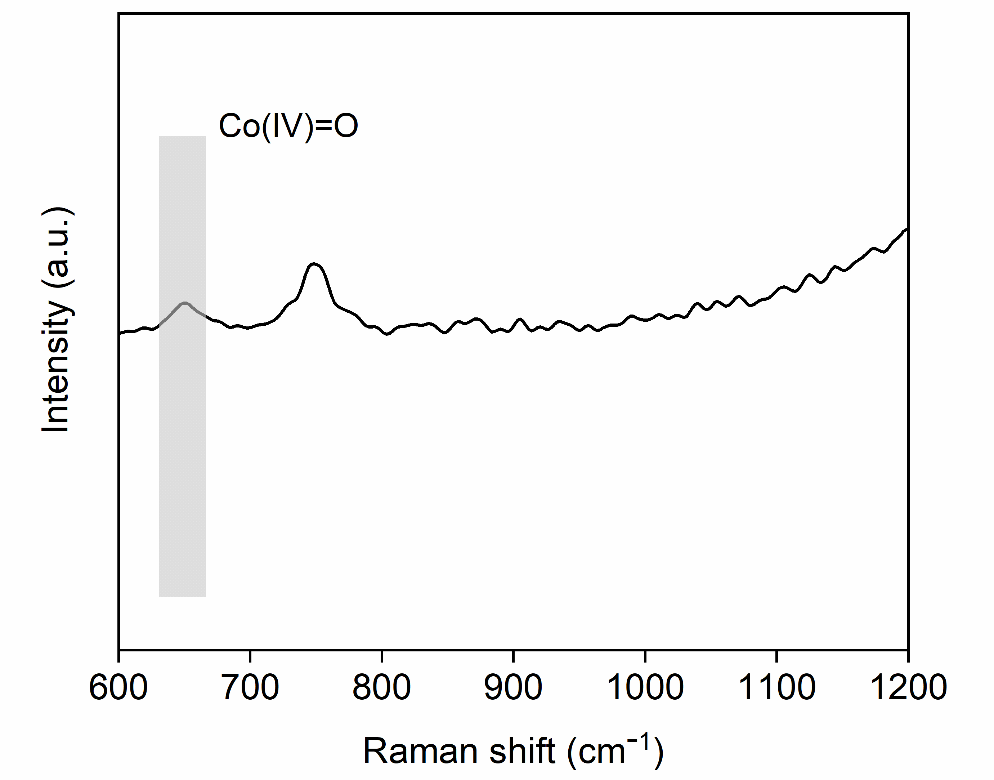


**Figure S21.** Raman spectra of CoN_4_/HClO system.


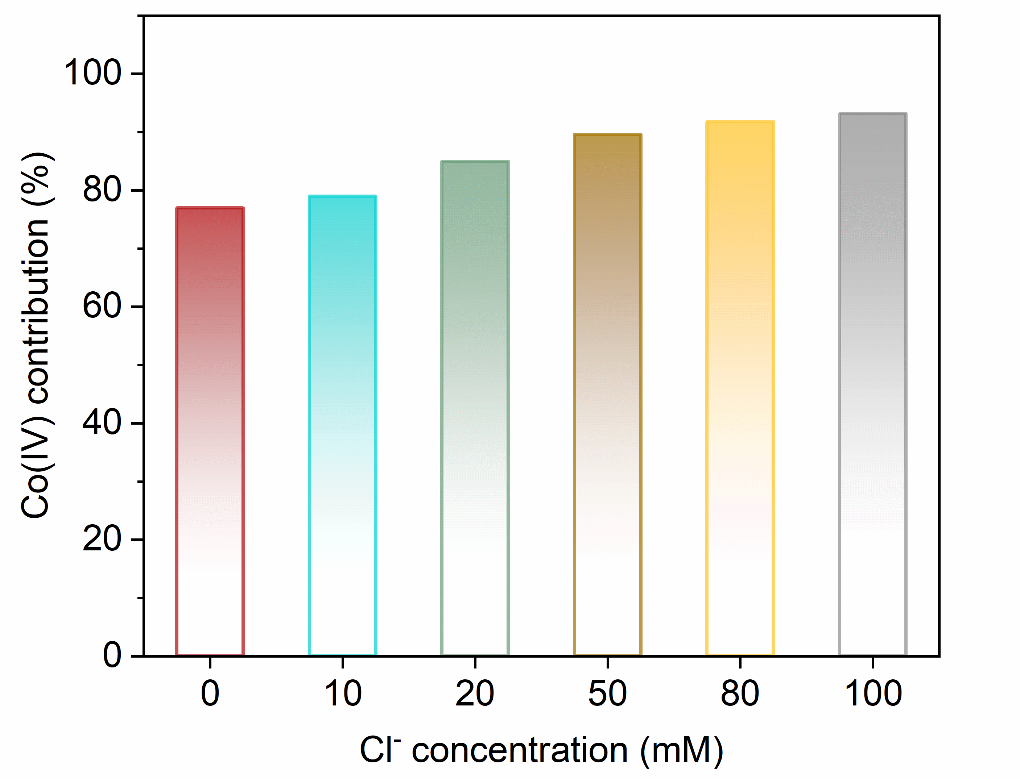


**Figure S22.** Co(IV)=O contribution ratio in PMS/Cl^−^/CoN_4_ with varied Cl^−^ concentration.


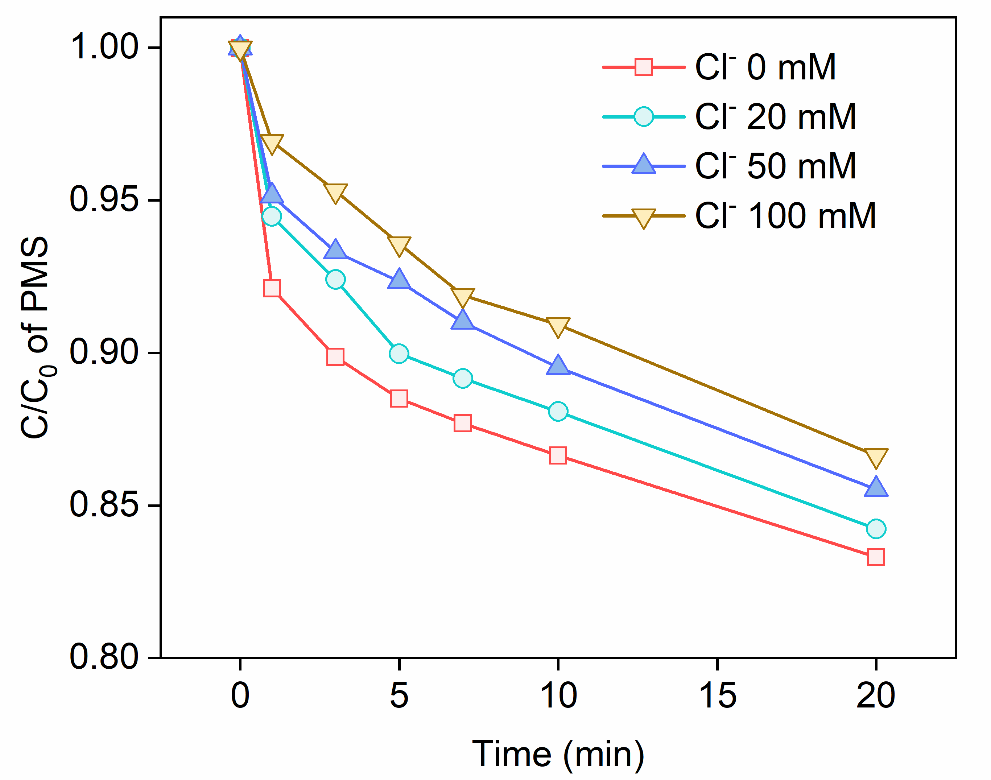


**Figure S23.** PMS consumption rates in PMS/Cl^−^/CoN_4_ with varied Cl^−^ concentration.


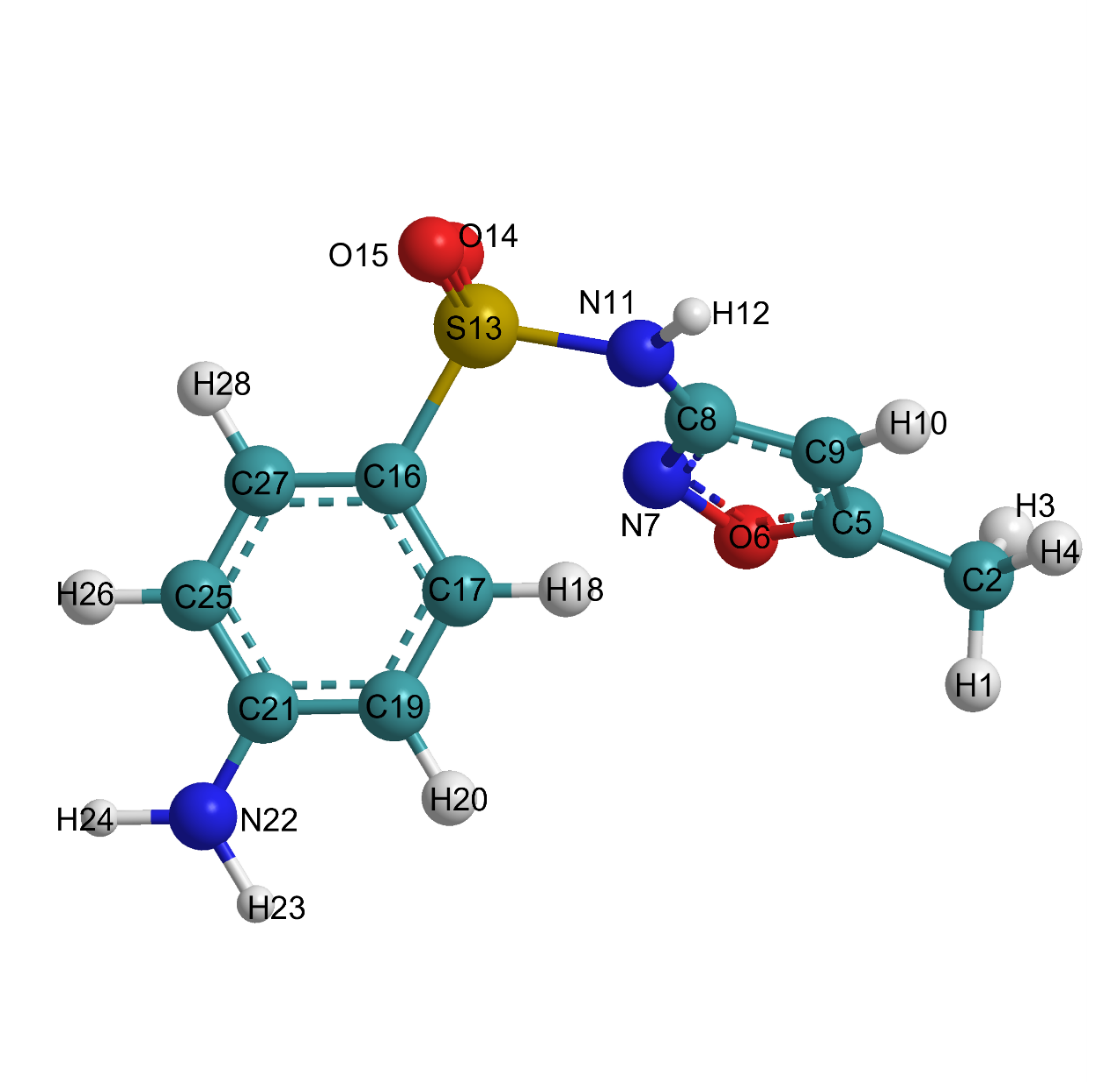


**Figure S24.** Optimized molecular structure of SMX.


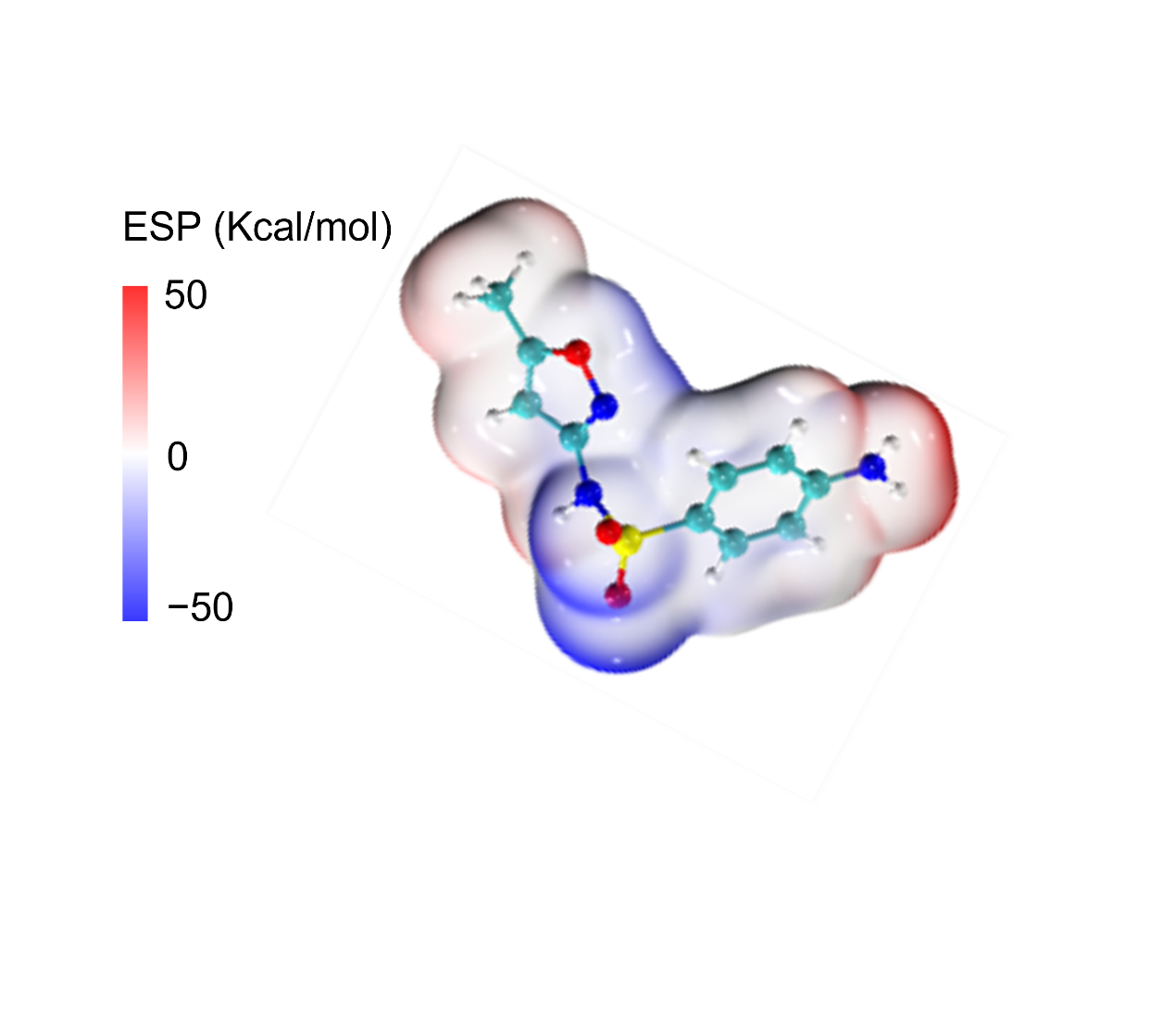


**Figure S25.** The ESP of the SMX molecular.


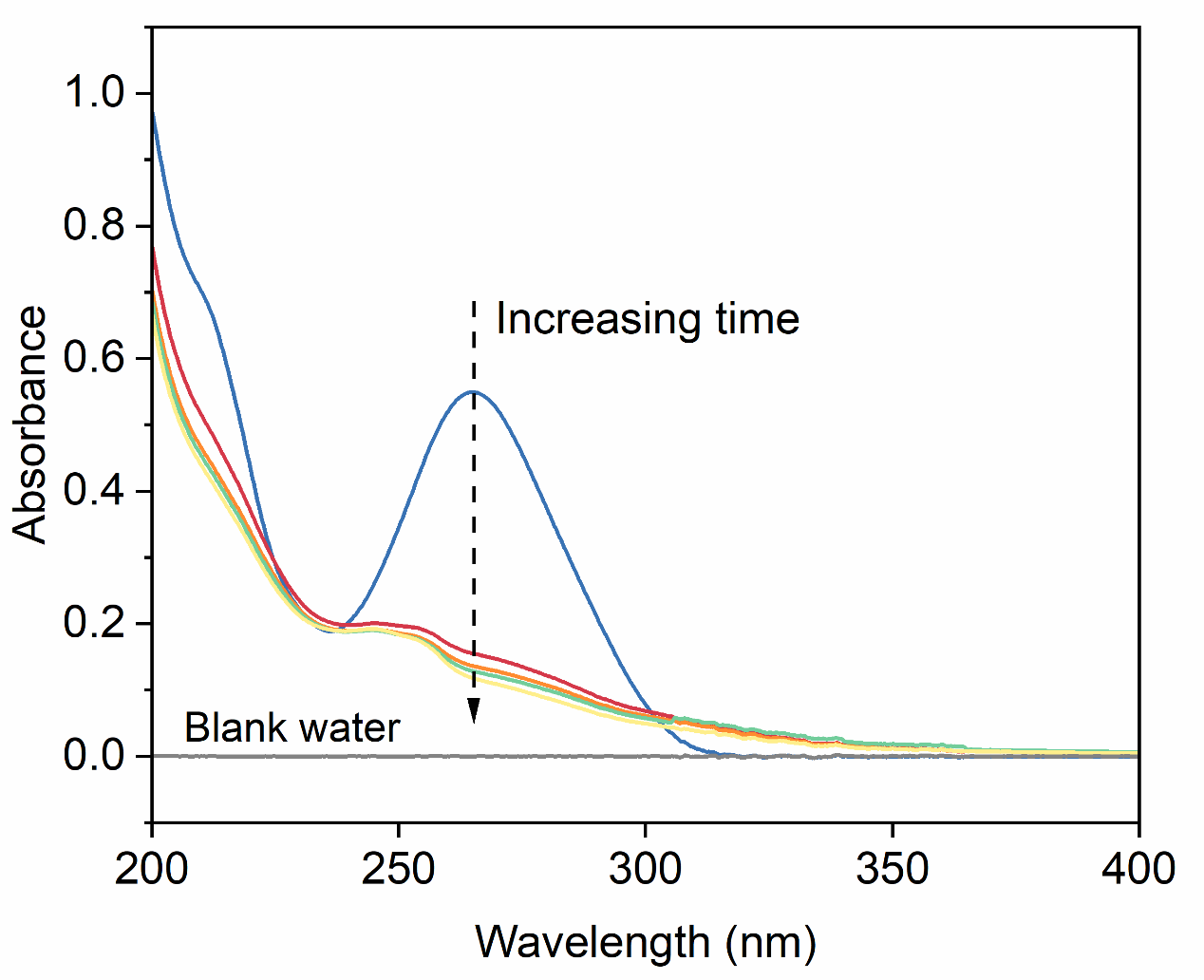


**Figure S26.** Time-dependent UV–vis spectra of SMX during degradation in the PMS/CoN_4_ system after methanol quenching.


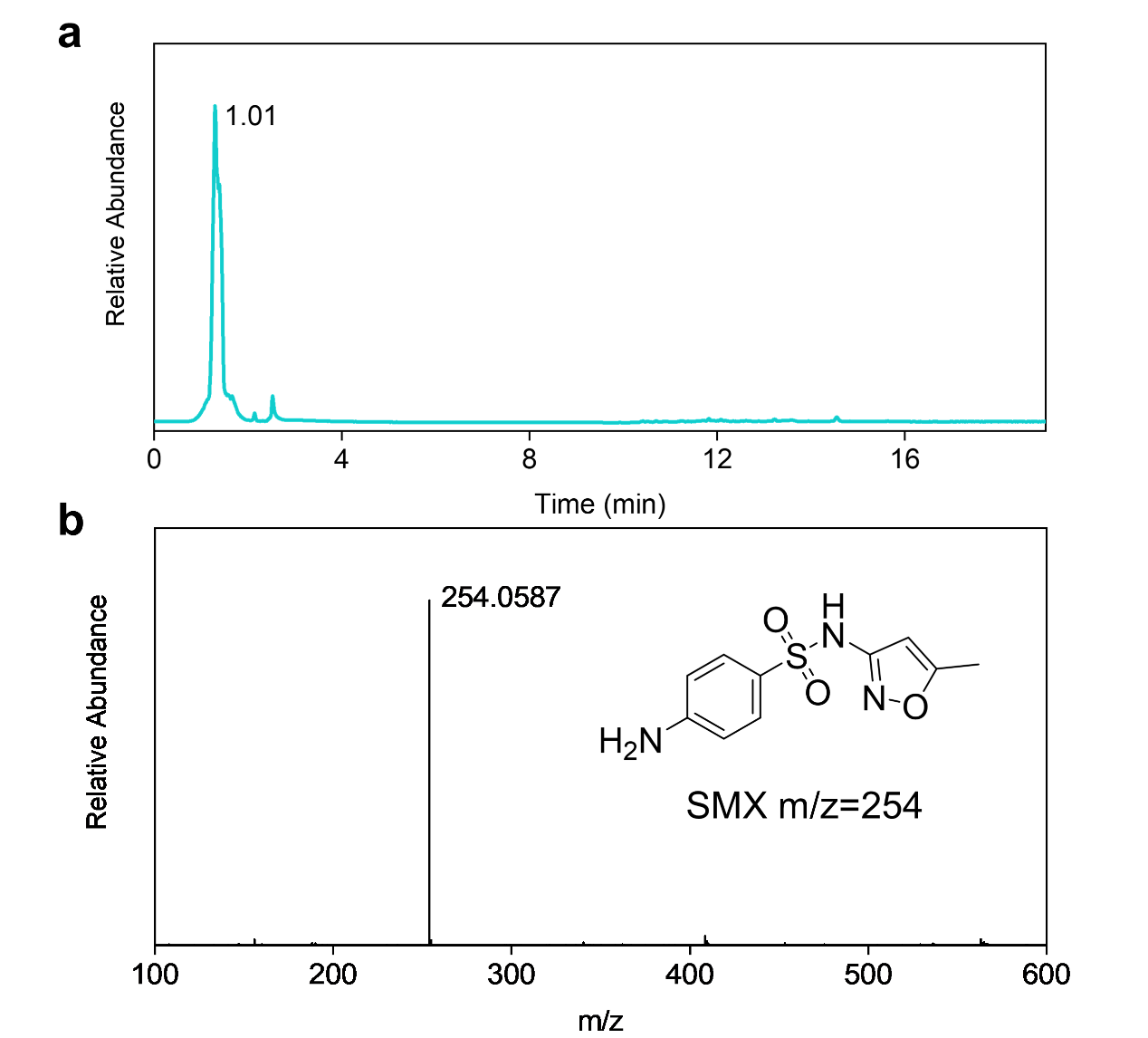


**Figure S27.** (a) Total ion chromatogram of the reaction solution before degradation obtained by LC–MS analysis. (b) MS spectra of the reaction solution before degradation.


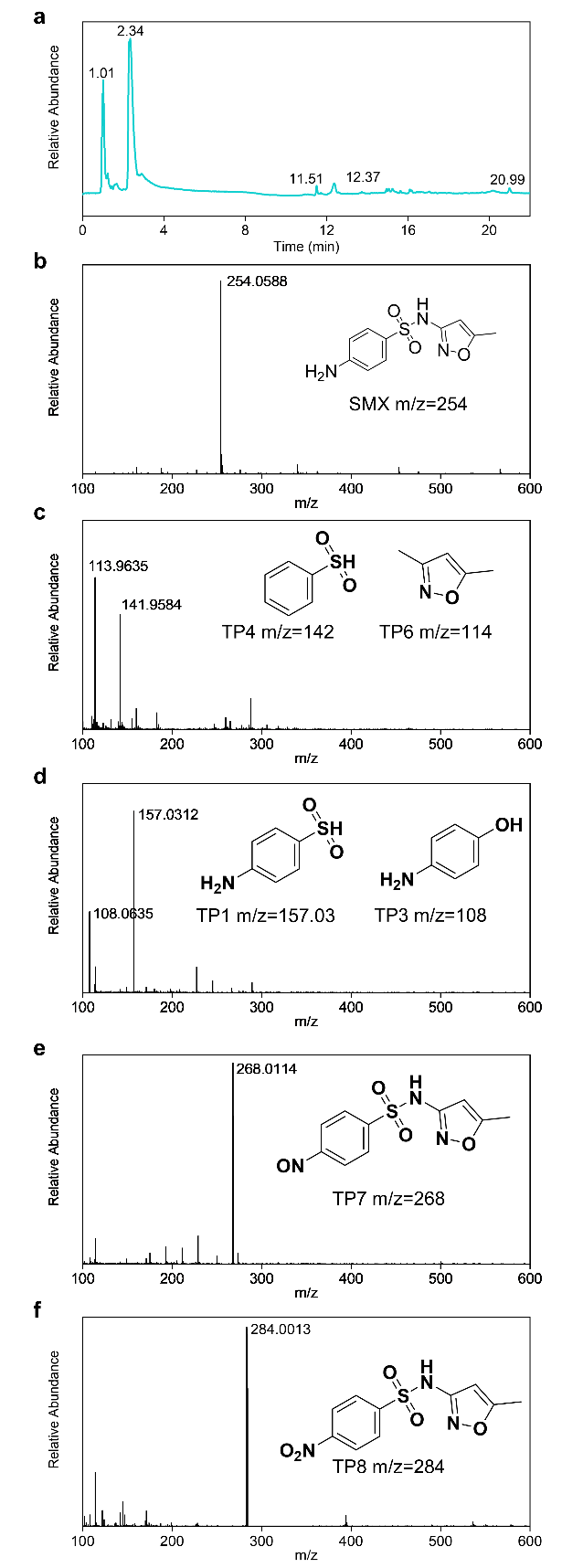


**Figure S28.** (a) Total ion chromatogram of the reaction solution after SMX degradation obtained by LC–MS analysis. MS spectra of degradation intermediates of SMX (b), TP4 and TP2 (c), TP1 and TP3 (d), TP7 (e) and TP8 (f).


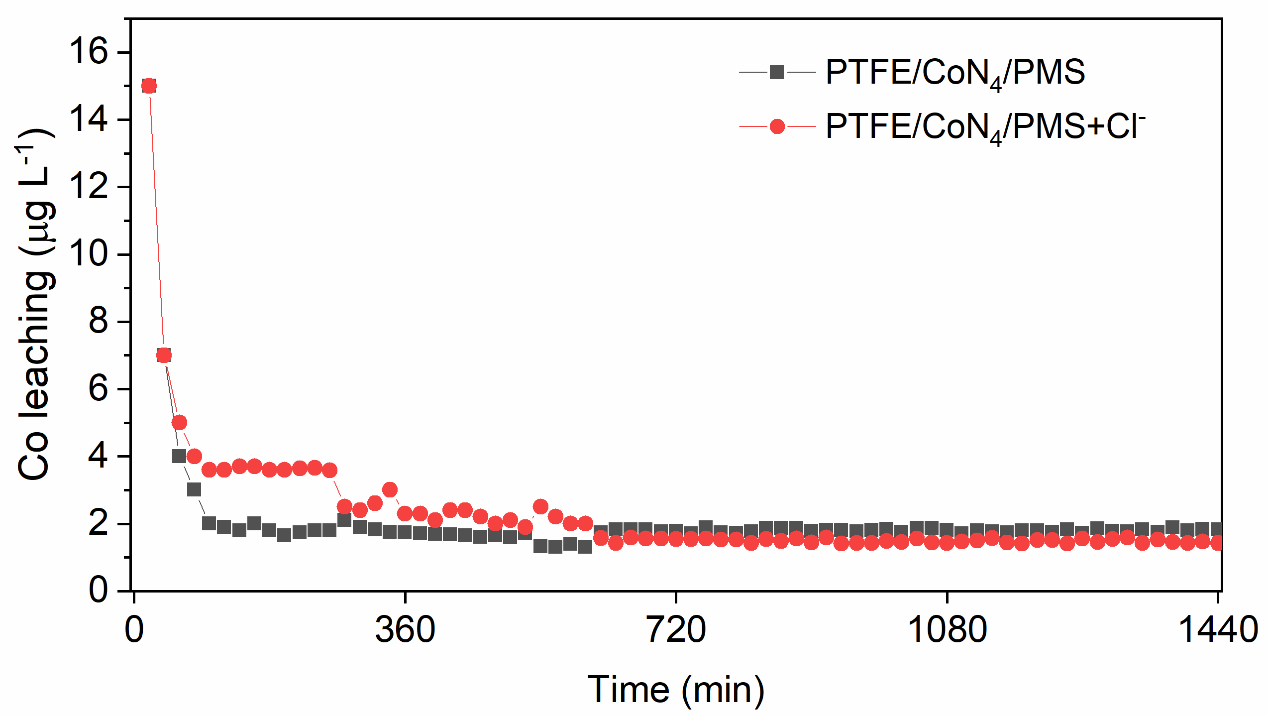


**Figure S29.** Co leaching efficiency using the SACs-loaded PTFE membrane.

# TABLE

**Table S1.** EXAFS fitting structural parameters of CoN_4_ at the Co K-edge.

| Sample | Shell | N^a^ | R(Å)^b^ | *σ*^2^(Å^2^)*^c^* | Δ*E*_0_(eV)*^d^* | *R* factor |
| --- | --- | --- | --- | --- | --- | --- |
| Co foil | Co-Co | 12* | 2.49 ± 0.01 | 0.0062 | 7.7 ± 0.3 | 0.0011 |
| Co-SACs | Co-N | 4.1 ± 0.3 | 1.88 ± 0.02 | 0.0069 | -5.6 ± 2.7 | 0.0097 |

*^a^CN*: coordination numbers; *^b^R*: bond distance; *^c^σ^2^*: Debye-Waller factors; *^d^ΔE_0_*: the inner potential correction. *R* factor: goodness of fit. *Ѕ*0^2^ was set to 0.78, according to the experimental EXAFS fit of Co foil reference by fixing CN as the known crystallographic value.

**Table S2.** Characteristics of real aqueous medium.

| Parameter (mg L^−1^) | Rain water | Lake water | Tap water |
| --- | --- | --- | --- |
| pH | 7.0 | 7.3 | 7.4 |
| COD_Mn_ | 1.1 | 7.1 | 0.6 |
| Cl^−^ | 150.7 | 32.7 | 36.7 |
| NO_3_^−^ | 50.1 | 15.6 | 13.2 |
| SO_4_^2−^ | 148.7 | 90 | 95 |
| K^+^ | 1.6 | 1.2 | 35.7 |
| Na^+^ | 40.1 | 20.3 | 40.3 |
| Mg^2+^ | 22.3 | 33.1 | 21.2 |

**Table S3.** HPLC parameters for the analysis of different pollutants.

| Pollutants | 0.1% acetic acid: acetonitrile | Wavelength/nm |
| --- | --- | --- |
| SMX | 70:30 | 265 |
| SMZ | 70:30 | 265 |
| SMR | 70:30 | 265 |
| SDZ | 70:30 | 265 |
| SPZ | 70:30 | 270 |
| SMM | 70:30 | 266 |
| SA | 70:30 | 258 |
| CIP | 80:20 | 278 |
| BPA | 70:30 | 276 |
| Ph | 80:20 | 280 |
| NB | 80:20 | 254 |
| BA | 80:20 | 230 |

**Table S4.** Comparison of catalytic performance of different catalysts in PMS-based systems.

| Catalyst | Cat.  dosage (g/L) | Oxidant | Oxidant  dosage  (mM) | | Contaminant | Contaminant dosage (mg/L) | *k*_obs_ | Ref. |
| --- | --- | --- | --- | --- | --- | --- | --- | --- |
| *Single-atom catalysts* | | | | | | | | |
| FeOF | 0.1 | H_2_O_2_ | | 10 | SMX | 20 | 0.27 | ^[10]^ |
| Co-N_3_O_1_ | 0.1 | PMS | | 1 | CIP | 5 | 0.29 | ^[11]^ |
| CoN_2+2_ | 0.2 | PMS | | 1.3 | CIP | 20 | 0.14 | ^[12]^ |
| Fe_1_-N_v_/CN | 0.5 | H_2_O_2_ | | 9.8 | CIP | 10 | 0.05 | ^[13]^ |
| Co-OCN | 0.03 | PMS | | 0.1 | APAP | 2 | 0.07 | ^[14]^ |
| FeCoO-g-C_3_N_4_ | 0.2 | PMS | | 0.8 | SMX | 10 | 0.09 | ^[15]^ |
| Co-g- C_3_N_4_ | 0.1 | PAA | | 0.2 | SMX | 2.5 | 0.15 | ^[16]^ |
| FeNO-GC-350 | 0.1 | PMS | | 0.4 | SMX | 10 | 0.24 | ^[17]^ |
| Co-N-CNT | 0.1 | PMS | | 1 | SMX | 10 | 0.16 | ^[18]^ |
| CoN_4_ | 0.05 | PMS | | 1 | SMX | 10 | 0.736 | This work |
| *Metal oxides* | | | | | | | | |
| BiVO_4_ | 0.32 | PMS | | 3 | CIP | 10 | 0.08 | ^[19]^ |
| Cu-Fe_2_O_3_ | 0.2 | H_2_O_2_ | | 10 | CIP | 10 | 0.05 | ^[20]^ |
| Co-Bi_2_O_2_CO_3_ | 0.6 | PMS | | 1 | SMX | 10 | 0.04 | ^[21]^ |
| OV-Co_3_O_4_ | 0.25 | Chlorite | | 0.1 | SMX | 6.3 | 0.13 | ^[22]^ |
| Cu-In_2_O_3_/OV | 0.5 | PMS | | 1 | CIP | 20 | 0.13 | ^[23]^ |
| CoCaAl-LDO | 0.05 | PMS | | 0.5 | CIP | 6.6 | 0.04 | ^[24]^ |
| *Carbon materials* | | | | | | | | |
| AC | 0.1 | PDS | | 5 | SMX | 10 | 0.07 | ^[25]^ |
| CNT | 0.1 | PDS | | 5 | SMX | 10 | 0.02 | ^[25]^ |

**Table S5.** Cyclic SMX degradation ratio and removal efficiency in PMS/CoN_4_ and PMS/Cl^−^/CoN_4_ systems.

|  | Degradation ratio in PMS/CoN_4_ | Removal efficiency in PMS/CoN_4_ | Degradation ratio in PMS/Cl^−^/CoN_4_ | Removal efficiency in PMS/Cl^−^/CoN_4_ |
| --- | --- | --- | --- | --- |
| Cycle 1 | 100 ± 0 | 100 | 92.0 ± 1.3 | 100 |
| Cycle 2 | 99.4± 1.73 | 99.4 | 91.54 ± 1.41 | 99.5 |
| Cycle 3 | 99.1 ± 1.27 | 99.1 | 90.53 ± 1.21 | 98.4 |
| Cycle 4 | 98.6 ± 1.58 | 98.6 | 89.79 ± 0.89 | 97.6 |
| Cycle 5 | 97.2 ± 0.92 | 97.2 | 89.24 ± 1.11 | 97.0 |
| Cycle 6 | 96.1 ± 2.1 | 96.1 | 89.06 ± 1.13 | 96.8 |
| Cycle 7 | 95.3± 1.70 | 95.3 | 87.49 ± 1.20 | 95.1 |
| Cycle 8 | 93.9 ± 2.10 | 93.9 | 87.12 ± 1.61 | 94.7 |
| Cycle 9 | 92.7 ± 1.13 | 92.7 | 87.49 ± 1.33 | 95.1 |
| Cycle 10 | 91.1 ± 1.20 | 91.1 | 85.74 ± 1.17 | 93.2 |

**Table S6.** Comparison of cycling durability of recent single-atom catalysts for pollutant degradation.

| Systems | Pollutants | Cycles | Degradation ratios in last cycle | Ref. |
| --- | --- | --- | --- | --- |
| Co_SA/Zn.O_-ZnO/PMS | SMX | 10 | ~90% | [26] |
| CoN_6_/C-P/PMS | APAP | 5 | 72.75% | [27] |
| Co1CNCl/S/PMS | Phenol | 4 | ~50% | [28] |
| FeN_4_/PMS | SMX | 5 | ~50% | [29] |
| Fe-Cu-CN/PMS | SMX | 5 | ~92% | [30] |
| CoN_4_/PMS | SMX | 10 | 91.1% | This work |
| CoN_4_/PMS/Cl^−^ | SMX | 10 | 93.2% | This work |

**Table S7.** Second-order reaction rate constants between different organics and ROS (M⁻¹ s⁻¹).

| Organics | Scavenged ROS | $k_{\cdot OH}$ | $k_{\mathrm{SO}_{4}^{\cdot-}}$ | $k_{1_{O2}}$ | $k_{\cdot O_{2}^{-}}$ | $k_{\mathrm{Fe}\left( \mathrm{IV} \right)=0}$ | Ref. |
| --- | --- | --- | --- | --- | --- | --- | --- |
| TBA | ·OH | 6.0×10^8^ | 4.0×10^5^ | 1.8×10^3^ | \ | 6.0×10 | ^[31]^ |
| MeOH | ·OH, SO_4_^·-^ | 9.7×10^8^ | 1.1×10^7^ | \ | 3.89×10^3^ | 5.72×10^2^ | ^[31]^ |
| DMSO | ·OH, SO_4_·^-^, Fe(IV)=O | 4.5×10^9^ | 3×10^9^ | 5.2×10^4^ | / | 1.3×10^5^ | ^[31]^ |
| FFA | ·OH, SO_4_·^-^, ^1^O_2_ | 15×10^10^ | 3.7×10^9^ | 1.2×10^8^ | 3.5×10^3^ | / | ^[31,32]^ |
| p-BQ | ·OH, ·O_2_^-^ | 6.6×10^9^ | / | ≈10^7^ | 0.9-1.0×10^9^ | / | ^[31,33,34]^ |
| BA | ·OH, SO_4_·^-^ | 4.2×10^9^ | 1.2×10^9^ | / | / | / | ^[31,35,36]^ |
| NB | ·OH | 3.9×10^9^ | <10^6^ | / | / | / | ^[31,35,36]^ |
| PMSO | ·OH, SO_4_·^-^, Co(IV)=O | 3.61×10^9^ | 3.17×10^8^ | / | / | 2.4×10^6^ | ^[31]^ |

**Table S8.** Ecotoxicity assessment of SMX and its transformation products towards fish, daphnid and green algae.

|  | TP98 | TP114 | TP157 | TP99 | TP108 | TP142 | TP284 | TP268 | SMX |
| --- | --- | --- | --- | --- | --- | --- | --- | --- | --- |
| 96-h LC50 | 2.52244 | 3.02119 | 3.123 | 2.43 | 2.22 | 3.85 | 3.06819 | 2.99607 | 2.42651 |
| ChV | 1.48001 | 1.95472 | 1.64 | 0.82 | 0.53 | 2.74 | 2.02119 | 1.95182 | 0.69897 |
| 48-h LC50 | 2.24797 | 2.72673 | 0.956 | 0.56 | 0.504 | 3.52 | 2.79169 | 2.72099 | 0.80821 |
| ChV | 1.16137 | 1.58546 | -1.07 | -1.44 | -1.48 | 2.29 | 1.69285 | 1.62839 | -1.16749 |
| 96-h EC50 | 2.00432 | 2.40483 | 1.59 | 1.14 | 1.05 | 3.07 | 2.53403 | 2.47276 | 1.33846 |
| ChV | 1.36361 | 1.7185 | 1.55 | 0.96 | 0.8 | 2.31 | 1.88195 | 1.82543 | 1.04532 |

# REFERENCES

**References:**

[1] J. Ding, H. Nie, S. Wang, Y. Chen, Y. Wan, J. Wang, H. Xiao, S. Yue, J. Ma and P. Xie, *Water Res.* **2021**, 189, 116605.

[2] F. N. Wires, *Comput. Molec. Sci.* **2012**, 2, 73−78.

[3] F. N. Wires, *Comput. Molec. Sci.* **2022**, 12, e1606.

[4] F. Weigend, R. Ahlrichs, *Phys. Chem. Chem. Phys.* 2005, 7, 3297.

[5] F. Weigend, *Phys. Chem. Chem. Phys.* **2006**, 8, 1057.

[6] S. Grimme, J. Antony, S. Ehrlich, H. Krieg, *J. Chem. Phys.* **2010**, 132, 154104.

[7] T. Lu, F. Chen, *J. Comput. Chem.* **2011**, 33, 580−592.

[8] T. Lu, *J. Phys. Chem. C* **2024**, 161.

[9] W. Humphrey, A. Dalke, K. Schulten. *J. Mol. Graph.* **1996**, 14, 33−38.

[10] D. Yu, L. Xu, K. Fu, S. Wang, M. Wu, W. Lu, C. Lv, J. Luo. *Nat. Commun.* **2024**, 15, 2241.

[11] Z. Wang, E. Almatrafi, H. Wang, H. Qin, W. Wang, L. Du, S. Chen, G. Zeng, P. Xu. *Angew. Chem. Int. Ed.* **2022**, 61, e202202338.

[12] X. Mi. *Angew. Chem. Int. Ed.* **2021**, 133, 46384643.

[13] L. Su, P. Wang, X. Ma, J. Wang, S. Zhan. *Angew. Chem. Int. Ed.* **2021**, 133, 21431−21436.

[14] Q.-Y. Wu, Z.-W. Yang, Z.-W. Wang, W.-L. Wang, *Proc. Natl. Acad. Sci.* **2023**, 120, e2219923120.

[15] S. Wang, Y. Liu, J. Wang, *Environ. Sci. Technol.* **2020**, 54, 10361−10369.

[16] B. Liu, W. Guo, W. Jia, H. Wang, Q. Si, N. Ren. *Environ. Sci. Technol.* **2021**, 55, 12640−12651.

[17] S. Wang, L. Xu, J. Wang. *Environ. Sci. Technol.* **2021**, 55, 15412−15422.

[18] J. Miao, Y. Zhu, J. Zhang, S. Cheng, B. Zhou, L. Zhang, PJJ. Alvarez, M. Long. *ACS Catal.* **2021**, 11, 9569−9577.

[19] F. Chen, G. Huang, Y. Zheng, Q. Zhao, H. Yu. *Water Res.* **2020**, 173, 115559.

[20] H. Zhan, R. Zhou, P. Wang, Q. Zhou. *Proc. Natl. Acad. Sci.* **2023**, 120, e2305378120.

[21] Q. Zhou, C. Song, P. Wang, Z. Zhao, Y. Li, S. Zhan. *Proc. Natl. Acad. Sci.* **2023**, 120, e2300085120.

[22] R. Su, Y. Gao, L. Chen, Y. Chen, N. Li, W. Liu, B. Gao, Q. Li. *Proc. Natl. Acad. Sci.* **2024***,* 121, e2319427121.

[23] Z. Zhao, P. Wang, C. Song, T. Zhang, S. Zhan, Y. Li. *Angew. Chem. Int. Ed.* **2023**, 62, e202216403.

[24] Z.-H. Xie, C. He, H. Zhou, L. Li, Y. Liu, Y. Du, W. Liu, Y. Mu, B. Lai. *Environ. Sci. Technol.* **2022**, 56, 8784-8795.

[25] J. Liang, K. Chen, X. Duan, H. Qiu, X. Xu, X. Cao. *Water Res.* **2022**, 224, 119113.

[26] Z.-Q. Zhang, P.-J. Duan, J.-X. Zheng, Y.-Q. Xie, C.-W. Bai, Y.-J. Sun, X.-J. Chen, F. Chen, H.-Q. Yu. *Nat. Commun.* **2025**, 16, 115.

[27] W.-M. Wang, Z.-W. Yang, D.-X. Wu, W.-L. Wang, Q.-Y. Wu. *Adv. Sci.* **2025**, 12, e12498.

[28] Z.-S. Zhu, Y. Wang, P. Wang, S. Zhong, H. Shi, J. Ren, I. Govindvijay, H. Sun, X. Duan, S. Wang. *Nat. Water* **2025**, 3, 211-221.

[29] J. Lu, A. Ding, L. Wang, W. Zhao, K. Zhang, M. Liu, X. Liu, Y. Li, Z. Li, D. Wu. *Small* **2026**, 22, e214930.

[30] L. Wang, Z. Guo, Y. Hou, B. Yang, L. Lei, J. Zhao, M. Qiu, Y. Li, Z. Li. *Angew. Chem. Int. Ed.* **2026**, 65, e202525747.

[31] C. Liu, J. Li, X. He, J. Yue, M. Chen, J. P. Chen, *Proc. Natl. Acad. Sci. USA* **2024**, 121, e2322283121.

[32] N. Li, H. Dai, M. He, J. Wang, Z. Cheng, B. Yan, W. Peng, G. Chen, *Engineering* **2024**, 168.

[33] M. N. Schuchmann, E. Bothe, J. von Sonntag, C. von Sonntag, J*. Chem. Soc., Perkin Trans.* *2* **1998**, 791.

[34] M. I. Gutierrez, *Photochem. Photobiol. Sci.* **2008**, 7, 480.

[35] Y. Shi, C. Su, Z. Sun, X. Xue, L. Li, X. Hu, *Sep. Purif. Technol.* **2025**, 129627.

[36] P. Neta, J. Grodkowski, *J. Phys. Chem. Ref. Data* **2005**, 34, 109.
